# Management of patients presenting to the emergency department with sudden onset severe headache: systematic review of diagnostic accuracy studies

**DOI:** 10.1136/emermed-2021-211900

**Published:** 2022-03-31

**Authors:** Matthew Walton, Robert Hodgson, Alison Eastwood, Melissa Harden, James Storey, Taj Hassan, Marc Stuart Randall, Abu Hassan, John Williams, Ros Wade

**Affiliations:** 1 Centre for Reviews and Dissemination, University of York, York, UK; 2 Department of Acute Internal Medicine, Leeds Teaching Hospitals NHS Trust, Leeds, UK; 3 Department of Emergency Medicine, Leeds Teaching Hospitals NHS Trust, Leeds, UK; 4 Department of Adult Neurology, Leeds Teaching Hospitals NHS Trust, Leeds, UK; 5 Patient representative, UK, UK

**Keywords:** emergency department, diagnosis, computed tomography, headache

## Abstract

**Objective:**

Advances in imaging technologies have precipitated uncertainty and inconsistency in the management of neurologically intact patients presenting to the Emergency Department (ED) with non-traumatic sudden onset severe headache with a clinical suspicion of subarachnoid haemorrhage (SAH). The objective of this systematic review was to evaluate diagnostic strategies in these patients.

**Methods:**

Studies assessing any decision rule or diagnostic test for evaluating neurologically intact adults with a severe headache, reaching maximum intensity within 1 hour, were eligible. Eighteen databases (including MEDLINE and Embase) were searched. Quality was assessed using QUADAS-2. Where appropriate, hierarchical bivariate meta-analysis was used to synthesise diagnostic accuracy results.

**Results:**

Thirty-seven studies were included. Eight studies assessing the Ottawa SAH clinical decision rule were pooled; sensitivity 99.5% (95% CI 90.8 to 100), specificity 24% (95% CI 15.5 to 34.4). Four studies assessing CT within 6 hours of headache onset were pooled; sensitivity 98.7% (95% CI 96.5 to 100), specificity 100% (95% CI 99.7 to 100). The sensitivity of CT beyond 6 hours was considerably lower (≤90%; 2 studies). Three studies assessing lumbar puncture (LP; spectrophotometric analysis) following negative CT were pooled; sensitivity 100% (95% CI 100 to 100), specificity 95% (95% CI 86.0 to 98.5).

**Conclusion:**

The Ottawa SAH Rule rules out further investigation in only a small proportion of patients. CT undertaken within 6 hours (with expertise of a neuroradiologist or radiologist who routinely interprets brain images) is highly accurate and likely to be sufficient to rule out SAH; CT beyond 6 hours is much less sensitive. The CT–LP pathway is highly sensitive for detecting SAH and some alternative diagnoses, although LP results in some false positive results.

**Key messages**

**What is already known on this subject**

- Guidelines typically recommend non-contrast CT head followed by lumbar puncture in patients who present with headache symptoms suspicious for subarachnoid haemorrhage.
- More recently, studies have questioned the need for routine lumbar puncture after a normal CT head.
- Additionally, a decision rule to direct imaging has been widely studied.

**What this study adds**

- In this systematic review and meta-analysis, we found that the Ottawa subarachnoid haemorrhage clinical decision rule has low specificity, and could result in significant additional unnecessary testing.
- CT head within 6 hours of headache onset, with images assessed by a neuroradiologist or radiologist who routinely interprets brain images, is highly accurate; around 658 CT-negative patients would have to undergo further investigation to identify a single case of subarachnoid haemorrhage.
- CT head undertaken beyond 6 hours is much less sensitive, therefore additional testing is more likely to be beneficial.
- In healthcare systems and settings in which neuroradiology expertise is unavailable, caution should be exercised when translating the diagnostic accuracy of CT head in the literature to clinical decision making.

**How this study might affect research, practice or policy**

- CT head within 6 hours of headache onset and with access to neuroradiology expertise is likely to be sufficient to rule out subarachnoid haemorrhage.
- The diagnostic accuracy of CT head may be contingent on time since symptom onset, which must be accounted for in practice, and investigated in future research.
- Risk tolerance of the patient and physician for the potential consequences of investigation and missed diagnoses will continue to inform practice.

## Introduction

Non-traumatic acute headache accounts for around 2% of adult Emergency Department (ED) attendances.^1^ Sudden onset severe headaches may be caused by a primary headache disorder or may be secondary to a more serious underlying pathology, such as subarachnoid haemorrhage (SAH). Diagnosis of SAH is particularly challenging in alert, neurologically intact patients presenting with acute severe headache. Clinical features separating these patients from higher volume complaints with a similar presentation (eg, migraine) are often unreliable indicators of who requires further investigation.^2^

Advances in imaging technologies have precipitated uncertainty and inconsistency in the optimal management of neurologically intact patients presenting to the ED with non-traumatic sudden onset severe headache.^3 4^ Given increasing evidence on the potentially low therapeutic value of lumbar puncture (LP) following CT of the head, and its associated adverse effects,^3 5–7^ updated evidence-based guidance is needed. We therefore undertook a systematic review of evidence on diagnostic strategies for neurologically intact adult patients presenting to hospital with non-traumatic sudden onset severe headache, reaching maximum intensity within 1 hour.

## Methods

The review protocol is registered on PROSPERO (CRD42020173265). This paper conforms to the recommendations of the Preferred Reporting Items for a Systematic Review and Meta-Analysis of Diagnostic Test Accuracy Studies statement.^8^

### Search strategy and selection criteria

Eighteen databases (including MEDLINE and Embase) were systematically searched in February 2020. Further details of the search strategy are presented in online supplemental file 1. To meet inclusion criteria, studies had to assess any care pathway for ruling out SAH (including clinical decision rules and specific diagnostic tests, such as CT or LP) in neurologically intact adult patients presenting to hospital with a sudden onset severe headache (reaching maximum intensity within 1 hour), with a clinical suspicion of SAH. Studies of patients who had suffered a head injury (ie, traumatic headache) were excluded. Any primary study design (other than single case study) was eligible for inclusion. Outcomes of interest included diagnostic accuracy, quality of life and adverse events. Two researchers (MW and RW) independently screened the titles and abstracts of all retrieved records and subsequently all full text publications for inclusion. Disagreements at each stage of the study selection process were resolved through discussion. Authors of potentially relevant conference abstracts were contacted for additional information. Relevant foreign language studies were translated and included in the review.

10.1136/emermed-2021-211900.supp1 Supplementary data

### Data extraction and quality assessment

Data were extracted on study methods, patient, intervention and reference standard characteristics, outcome measures, adverse events and results (presented in online supplemental file 2). Data extraction and quality assessment were undertaken by one researcher and independently checked by a second. The majority of studies were assessed for quality using the Quality Assessment of Diagnostic Accuracy Studies 2 (QUADAS-2) tool.^9^ The QUADAS-2 tool was not appropriate for studies where a reference standard test was not used, therefore, a quality assessment tool was developed by RW specifically for the review, piloted and refined before use (see online supplemental file 3 for details).

10.1136/emermed-2021-211900.supp2 Supplementary data

10.1136/emermed-2021-211900.supp3 Supplementary data

### Data analysis

Where sufficient information was reported, diagnostic accuracy data were extracted into 2×2 tables to calculate sensitivity, specificity, false positive and false negative rates. Where equivalent diagnostic strategies or tools were used in three or more studies, the hierarchical bivariate model described by Reitsma *et al* 10 was fitted, along with an extension described by Simmonds and Higgins^11^ to meta-analyse sensitivity and specificity while accounting for correlation between the two, and within-person correlation between test results. Meta-analyses used standard random-effects DerSimonian-Laird methods. Subgroups were analysed separately to account for underlying differences in diagnostic strategies. The diagnostic accuracy of CT conducted <6 hours from headache onset was analysed separately, as CT accuracy is known to drop rapidly outside of this time frame.^12^ The accuracy of different methods of cerebrospinal fluid (CSF) analysis was also assessed. Where results could not be pooled, they were synthesised narratively along with reported adverse event data.

### Public and patient involvement

A patient collaborator with experience of presenting to an ED with a sudden onset severe headache was involved throughout the project. Three additional patients were recruited to an advisory group. The patients provided input during protocol development and interpretation of review findings.

## Results

**Figure 1:**
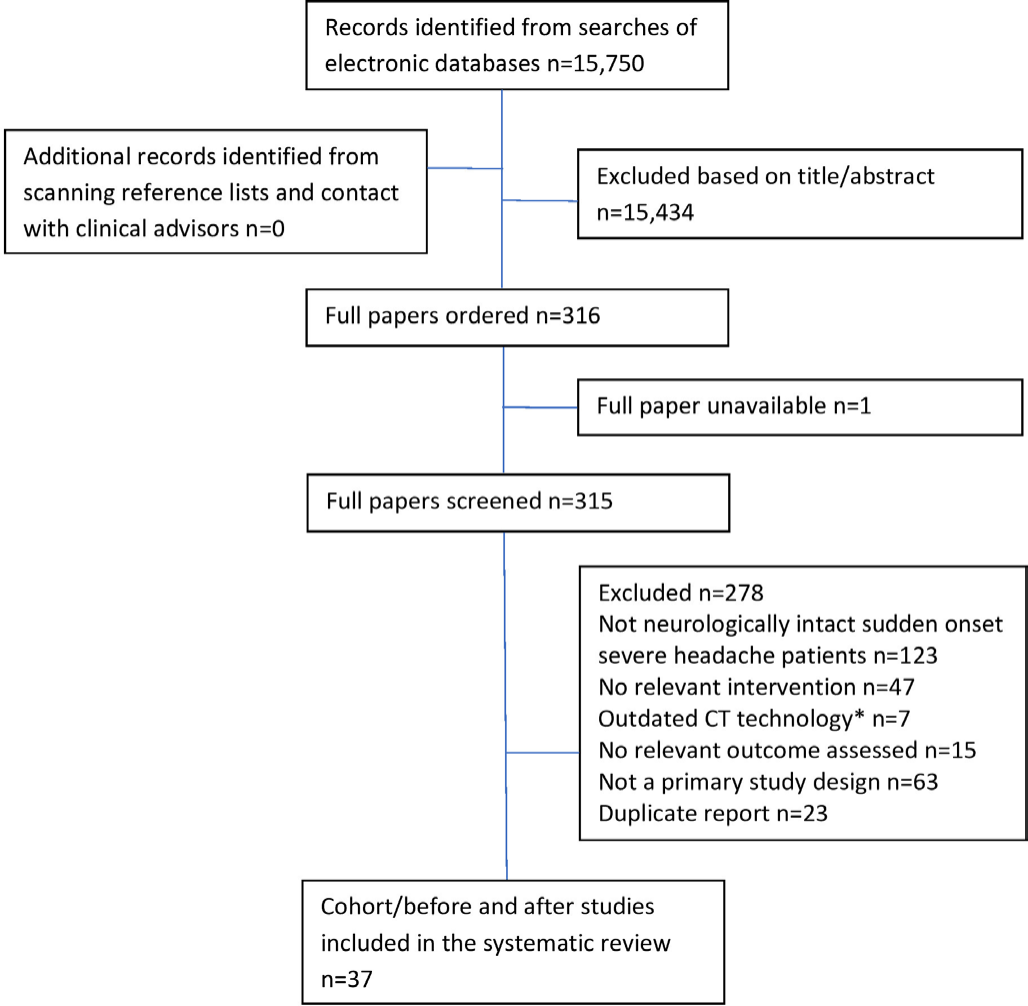
Flow diagram of the study selection process. *Any study which recruited patients before the year 2000 was considered to have used outdated CT technology.

The search strategy identified 15 750 records; 37 cohort/before and after studies were eligible for inclusion (figure 1 and table 1). More detailed study characteristics and results are presented in online supplemental file 2.

**Table 1:** Studies included in the systematic review

Intervention	Study	Location	N	Study design
Clinical decision rules (Canadian clinical decision rules 1, 2, 3; Ottawa SAH Rule)	Bellolio *et al* 13	USA	454	Retrospective cohort
	Cheung *et al* 14	Hong Kong	500	Retrospective cohort
	Chu *et al* 15	Australia	137	Retrospective cohort (substudy of a prospective cohort)
	Kelly *et al* 16	Australia	59	Retrospective cohort
	MacDonald *et al* 17	UK	280	Retrospective cohort
	Matloob *et al* 18	UK	112	Retrospective cohort
	Pathan *et al* 19	UK	145	Retrospective cohort
	Perry *et al* 20	Canada	1999	Prospective cohort
	Perry *et al* 21	Canada	2131	Prospective cohort
	Perry *et al* 22	Canada	1153; overlap with Perry *et al* 23	Prospective cohort
	Perry *et al* 23	Canada	3672	Prospective before/after
	Wu *et al* 24	Taiwan	913	Retrospective cohort
	Yiangou *et al* 25	UK	162	Retrospective cohort
CT–LP pathway	Blok *et al* 26	The Netherlands	760	Retrospective cohort
	Cooper *et al* 7	UK	517	Retrospective cohort
	Dutto *et al* 27	Italy	70	Before/After
	Perry *et al* 28	Canada	891	Retrospective cohort
	Perry *et al* 29	Canada	592	Prospective cohort
	Valle Alonso *et al* 30	Spain	74	Retrospective cohort
CT	Austin *et al* 31	UK	250	Retrospective cohort
	Backes *et al* 32	The Netherlands	250	Retrospective cohort
	Blok *et al* 26	The Netherlands	760	Retrospective cohort
	Cooper *et al* 7	UK	517	Retrospective cohort
	Khan *et al* 33	Canada	2412; overlap with Perry *et al* 12	Prospective cohort (secondary analysis)
	Perry *et al* 20	Canada	1999; overlap with Perry *et al* 12	Prospective cohort
	Perry *et al* 12	Canada	3132	Prospective cohort
	Perry *et al* 23	Canada	1204 had CT <6 hours	Prospective before/after
	Valle Alonso *et al* 30	Spain	85	Retrospective cohort
LP	Brunell *et al* 34	Sweden	453	Retrospective cohort
	Cooper *et al* 7	UK	309 had LP	Retrospective cohort
	Dupont *et al* 35	USA	117 had LP	Retrospective cohort
	Gangloff *et al* 36	Canada	706	Retrospective cohort
	Heiser *et al* 37	USA	676	Retrospective cohort
	Horstman *et al* 38	The Netherlands	30	Retrospective cohort
	Migdal *et al* 39	USA	245	Retrospective cohort
	Perry *et al* 40	Canada	220	Prospective cohort (substudy)
	Perry *et al* 41	Canada	1739	Prospective cohort (substudy)
	Sansom *et al* 42	UK	60	Retrospective cohort
	Valle Alonso *et al* 30	Spain	74 had LP	Retrospective cohort
CTA	Alons *et al* 44	The Netherlands	70	Retrospective cohort
	Alons *et al* 45	The Netherlands	88	Retrospective cohort and meta-analysis
History and examination	Locker *et al* 2	UK	353	Retrospective cohort
	Perry *et al* 46	Canada	747	Prospective cohort
	Backes *et al* 47	The Netherlands	247	Retrospective cohort

CSF, cerebrospinal fluid; CTA, CT angiography; LP, lumbar puncture; SAH, subarachnoid haemorrhage.

**Figure 2:**
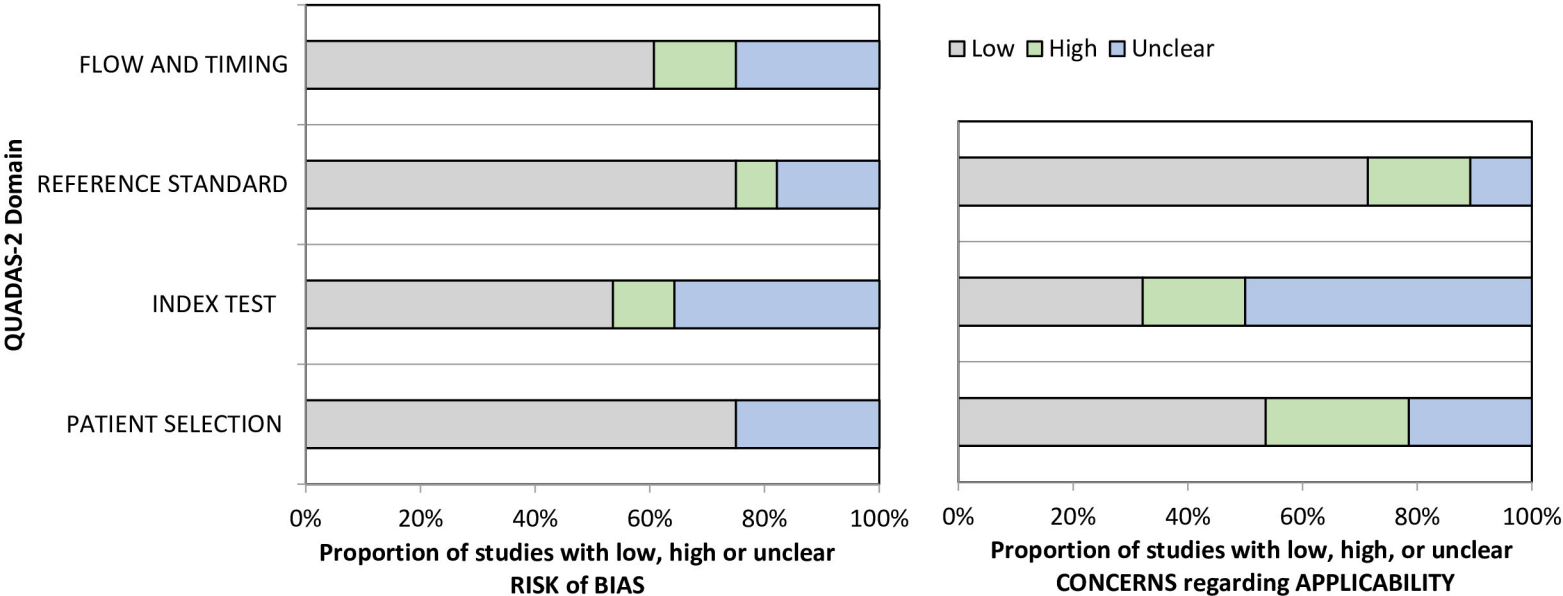
Quality Assessment of Diagnostic Accuracy Studies 2 (QUADAS-2) results.

Twelve studies had a low risk of bias for all domains, the other 25 were at risk of bias. Twenty-eight studies were assessed using the QUADAS-2 tool; results are summarised in figure 2.^9^ Nine studies did not use a reference standard test, therefore, QUADAS-2 was inappropriate; a quality assessment tool developed specifically for the review was used instead. Quality assessment results are presented in the online supplemental file 3.

### Clinical decision rules

Thirteen studies assessed the clinical decision rules developed by Perry *et al* for screening patients according to the presence of clinical characteristics associated with a high risk of SAH.^13–25^ The predecessors of the Ottawa SAH Rule (sometimes termed the ‘Canadian clinical decision rules 1, 2 and 3’) were evaluated in six studies. Results of these studies can be found in online supplemental file 2. Rule 1 was refined to develop the final Ottawa SAH Rule, which states that alert patients with new severe atraumatic headache, reaching maximum intensity within 1 hour, require investigation if one of the following are present: age ≥40 years, neck pain/stiffness, witnessed loss of consciousness, onset during exertion, thunderclap headache or limited neck flexion.^21^

A summary of the diagnostic performance of the Ottawa SAH Rule in the individual studies and pooled results generated from the bivariate meta-analysis are presented in table 2. Perry *et al* (2017) is excluded,^22^ due to patient overlap with the larger Perry *et al* (2020) study.^23^ The overall SAH prevalence in the studies ranged from 1.6%^24^ to 10%^14^ with a population-weighted mean prevalence of 5.0%. The Ottawa SAH Rule is highly sensitive, but specificity was low; strict application of the rule would result in 76% of SAH-negative patients undergoing further investigation with no additional benefit. There was considerable heterogeneity in false positive rates (FPR), potentially due to study population differences or inconsistent application of the rule. No studies assessed the accuracy of the Ottawa SAH Rule in patient subgroups by time to headache peak.

**Table 2:** Diagnostic performance of Ottawa SAH Rule

Study	N	Sens (%)	95% CI	Spec (%)	95% CI	FNR (%)	95% CI	FPR (%)	95% CI
Perry *et al* 21	2131	100	100 to 100	15.3	13.7 to 16.8	0.0	0.0 to 0.0	84.7	83.2 to 86.3
Bellolio *et al* 13	454	100	100 to 100	7.6	5.17 to 10.1	0.0	0.0 to 0.0	92.4	89.9 to 94.8
Yiangou *et al* 25	162	100	100 to 100	38.7	31.4 to 46.6	0.0	0.0 to 0.0	61.0	53.4 to 68.6
Cheung *et al* 14	500	94.0	87.4 to 100	32.9	28.5 to 37.2	6.0	0.0 to 12.6	67.1	62.8 to 71.5
Chu *et al* 15	137	100	100 to 100	22.4	15.3 to 29.4	0.0	0.0 to 0.0	77.6	70.6 to 84.7
Pathan *et al* 19	145	100	100 to 100	44.3	36.1 to 52.5	0.0	0.0 to 0.0	55.7	47.5 to 63.9
Wu *et al* 24	913	100	100 to 100	37.0	33.8 to 40.1	0.0	0.0 to 0.0	63.0	59.9 to 66.2
Perry *et al* 23	3672	100	100 to 100	12.7	11.6 to 13.9	0.0	0.0 to 0.0	87.3	86.1 to 88.4
Pooled (n=8)	8114	99.5	90.8 to 100	23.7	15.5 to 34.4	0.49	0.00 to 9.2	76.3	65.6 to 84.5

FNR, false negative rate; FPR, false positive rate; N, number; Sens, sensitivity; Spec, specificity.

### Pathway of CT followed by LP

The pathway of non-contrast CT followed by LP was assessed in six studies.^7 26–30^ Only one reported complete diagnostic data, so meta-analysis was not performed. Overall, the pathway was highly sensitive, but specificity was low in some studies owing to the high FPR for LP. Importantly, this pathway also identified other significant pathologies, such as intracerebral haemorrhage, brain tumour and meningitis. More detailed results for this pathway can be found in online supplemental file 2.

### Computed tomography

The diagnostic accuracy of CT was assessed in nine studies,^7 12 20 23 26 30–33^ although three studies had significant patient overlap,^12 20 33^ therefore, only the results for the largest of the three are presented.^12^

#### CT undertaken within 6 hours of headache onset

Four studies of CT <6 hours from headache onset were included in bivariate meta-analysis (table 3).^12 23 30 32^ In all four studies, CT scans were assessed by neuroradiologists or radiologists who routinely interpret head CT images. Perry *et al* (2020) classed two incidental aneurysms with traumatic tap on subsequent LP as SAH, and thus as false negatives. This is inconsistent with the other included studies and with our interpretation of what constitutes a false negative. Therefore, these two patients were reclassified as true negatives.

**Table 3:** Diagnostic performance of CT (<6 hours from headache onset)

Study	N	Sens (%)	95% CI	Spec (%)	95% CI	FNR (%)	95% CI	FPR (%)	95% CI
Perry *et al* 12	953	100	100 to 100	100	100 to 100	0.0	0.0 to 0.0	0.0	0.0 to 0.0
Backes *et al* 32	135	100	100 to 100	100	100 to 100	0.0	0.0 to 0.0	0.0	0.0 to 0.0
Valle Alonso *et al* 30	85	100	100 to 100	98.7	96.1 to 100	0.0	0.0 to 0.0	1.3	0.0 to 3.9
Perry *et al* (reclassified)^23^	1204	97.2	94.2 to 100	100	100 to 100	2.8	0.0 to 5.8	0.0	0.0 to 0.0
Pooled (n=4)	2377	98.7	96.5 to 100	100	99.7 to 100	1.34	0.50 to 3.52	0.00	0.00 to 0.34

FNR, false negative rate; FPR, false positive rate; N, number; Sens, sensitivity; Spec, specificity.

The recruitment of patients from SAH patient databases in Backes *et al* 32 meant that SAH patients were over-represented in the study population (41.5%). SAH prevalence ranged from 9.2%^23^ to 12.7%^12^ in the other three studies, with a population-weighted average prevalence of 10.8%. Assuming that these patients are representative of those presenting to EDs in practice, the pre-test probability of SAH in patients with headache who undergo CT within 6 hours is 10.8%. Using the pooled estimate of diagnostic accuracy, the post-test probability of having suffered a SAH after a negative <6 hour CT result is 0.15%. Assuming a hypothetical follow-up test (eg, LP) has 100% accuracy, this means that 658 (95% CI 250 to 1749) patients would have to undergo further investigation to identify a single case of SAH.

One additional study assessed the diagnostic accuracy of CT <6 hours, but was excluded from the meta-analysis as it did not report sufficient diagnostic accuracy data to construct a 2×2 table to calculate sensitivity and specificity.^26^ In this study, 760 patients had a negative CT (assessed by a staff radiologist) and subsequently underwent LP; 7% of CSF samples were initially considered positive for SAH, but subarachnoid blood was identified in only one patient on review by two neuroradiologists and a neurologist. The negative predictive value for detection of blood on CT by staff radiologists was 99.9% (95% CI 99.3 to 100).

#### CT undertaken at any time interval from headache onset

Three studies of CT undertaken at any time interval from headache onset were included in bivariate meta-analysis (table 4).^7 12 32^ In all three studies, CT scans were assessed by neuroradiologists or radiologists who routinely interpret head CT images. The prevalence of SAH in patients undergoing CT at any time since headache onset was lower than in those who underwent CT within 6 hours. Prevalence was 2.7% in the study by Cooper *et al* 7 and 7.7% in the study by Perry *et al.* 12 As noted above, SAH patients were over-represented in the Backes *et al* study population (35.2%).^32^

**Table 4:** Diagnostic performance of CT (at any time)

Study	N	Sens (%)	95% CI	Spec (%)	95% CI	FNR (%)	95% CI	FPR (%)	95% CI
Perry *et al* 12	3132	92.9	89.7 to 96.2	100	100 to 100	7.08	3.8 to 10.3	0.00	0.0 to 0.0
Backes *et al* 32	247	97.6	94.4 to 100	100	100 to 100	2.38	0.0 to 5.6	0.00	0.0 to 0.0
Cooper *et al* 7	510	92.9	79.4 to 100	100	100 to 100	7.14	0.0 to 20.6	0.00	0.0 to 0.0
Pooled (n=3)	3889	94.1	91.0 to 96.2	100	100 to 100	5.92	3.85 to 8.99	0.00	0.00 to 0.00

FNR, false negative rate; FPR, false positive rate; N, number; Sens, sensitivity; Spec, specificity.

The pooled sensitivity of CT at any time since headache onset was 94.1% (95% CI 91.0 to 96.2). This result includes patients who had CT <6 hours, as well as CT >6 hours, from symptom onset. Results from Perry *et al* 12 and Backes *et al* 32 suggest CT scans performed >6 hours after symptom onset have significantly poorer performance, reporting sensitivities of 85.7% (95% CI 78.3 to 90.9) and 90.0% (95% CI 76.3 to 97.2), respectively. The bimodal nature of the diagnostic performance of CT means that the ‘CT at any time’ statistics are misleading, as the timing of CT has a significant impact on the pre-test and post-test probabilities of SAH.

One additional CT study compared interpretation by emergency physicians (images viewed on standard resolution desktop screens) with the reference standard of neuroradiologists’ readings (images viewed using dedicated high definition screens).^31^ The sensitivity of CT interpreted by emergency physicians was 84% (95% CI 63.9 to 95.5) and specificity was 95% (95% CI 90.9 to 97.2). However, this study was considered to have a high risk of bias due to the difference in hardware used between the two specialties for examining CT images.

### Lumbar puncture

The diagnostic accuracy of LP in patients judged to be SAH-negative using CT was assessed in 11 studies.^7 30 34–42^ The method of assessing CSF for xanthochromia varied, with Canadian and American studies predominantly using visual inspection and UK and European studies predominantly using spectrophotometry. LP was not always undertaken ≥12 hours from symptom onset. The standard UK NHS practice is to take the CSF sample ≥12 hours from symptom onset to allow xanthochromia to develop, with samples analysed using spectrophotometry.^43^

#### Spectrophotometric CSF analysis

Three studies reported diagnostic accuracy data for spectrophotometric CSF analysis following negative CT (table 5).^7 36 40^ Samples were analysed for presence of bilirubin using the UK National External Quality Assessment Service protocol/assay.^43^ The prevalence of SAH in these studies was only 0.65%, likely due to prescreening with CT. The FPR (and subsequent rate of angiography) was particularly high in Perry *et al* (2006), perhaps due to reported limitations in the spectrophotometric equipment used by the authors. The FPR in the more recent studies was substantially lower and likely better represents the diagnostic accuracy of CSF spectrophotometry in current practice.

**Table 5:** Diagnostic performance of spectrophotometric CSF inspection (UK National External Quality Assessment Service)

Study	N	Sens (%)	95% CI	Spec (%)	95% CI	FNR (%)	95% CI	FPR (%)	95% CI
Perry *et al* 40	220	100	100 to 100	83.0	78.0 to 88.0	0.0	0.0 to 0.0	17.0	12.0 to 22.0
Gangloff *et al* 36	706	100	100 to 100	98.1	96.8 to 99.1	0.0	0.0 to 0.0	1.9	0.9 to 2.9
Cooper *et al* 7	309	100	100 to 100	96.8	94.8 to 98.7	0.0	0.0 to 0.0	3.3	0.1 to 5.2
Pooled (n=3)	1235	100	100 to 100	95.2	86.0 to 98.5	0.00	0.00 to 0.00	4.78	1.52 to 14.0

FNR, false negative rate; FPR, false positive rate; N, number; Sens, sensitivity; Spec, specificity.

Three further studies assessed CSF spectrophotometry in patients who underwent LP after negative CT, but reporting was insufficient for meta-analysis.^34 38 42^ Horstman *et al* included 30 patients with a negative CT result for whom bilirubin was detected in the CSF; aneurysms were identified in 13 patients; however, all cases presented 4–14 days after symptom onset.^38^ Brunell *et al* included 453 patients, 400 (88%) of whom presented with thunderclap headache; 14 (3%) patients had a pathological diagnosis based on LP, most commonly aseptic meningitis, and 5 (1.1%) had SAH.^34^ Four of the five SAH patients had non-aneurysmal SAH which did not require surgical intervention and the other SAH patient had reduced consciousness, therefore did not strictly meet the inclusion criteria for this review.^34^ Sansom *et al* included 60 CT-negative patients with thunderclap headache; all samples were negative for xanthochromia but 8/60 CSF examinations were abnormal for other CSF parameters (protein, glucose, cells, microscopy), with cerebral infarction confirmed in two of these patients on subsequent investigation.^42^

#### Visual CSF inspection

Five studies examined the diagnostic accuracy of visible xanthochromia in CT-negative patients with further investigation and follow-up used as a reference standard.^35 36 39–41^ Three studies included sufficient information to calculate diagnostic accuracy (table 6). Sensitivity varied widely (50%–93%), due to the low prevalence of SAH (2%). The pooled false negative rate of 15% for visual inspection was higher than that for spectrophotometric analysis (0%).

**Table 6:** Diagnostic performance of visual CSF inspection across identified studies

Study	N	Sens (%)	95% CI	Spec (%)	95% CI	FNR (%)	95% CI	FPR (%)	95% CI
Perry *et al* 40	220	50.0	0.0 to 100	96.8	94.4 to 99.1	50.0	0.0 to 100	3.21	0.9 to 5.6
Dupont *et al* 35	117	92.9	79.4 to 100	95.1	91.0 to 99.3	7.1	0.0 to 20.6	4.85	0.7 to 9.0
Gangloff *et al* 36	706	80.0	44.9 to 100	98.7	97.9 to 99.5	20.0	0.0 to 55.1	1.28	0.5 to 2.1
Pooled (n=3)	1043	84.9	60.0 to 95.5	97.6	95.3 to 98.8	15.1	4.5 to 40.1	2.43	1.23 to 4.75

FNR, false negative rate; FPR, false positive rate; N, number; Sens, sensitivity; Spec, specificity.

Migdal *et al* assessed 245 patients with ‘low risk clinical features’, which aligned with the population in this review, but identified no cases of SAH. However, 13/245 (5.3%) patients had LP-related complications that resulted in a return visit to the ED or hospitalisation.^39^ Perry *et al* examined the diagnostic accuracy of visible xanthochromia in ‘abnormal’ CSF samples drawn from 1739 (mostly) CT-negative patients; there were 15 (0.9%) patients classed as having aneurysmal SAH, 7 of whom had visible xanthochromia in their CSF.^41^

#### Red blood cell-based CSF analysis thresholds

Two studies explored methods to distinguish SAH from ‘traumatic tap’, where blood enters the CSF sample due to the LP procedure itself. Perry *et al* found that the presence of fewer than 2000×10^6^/L red blood cells (RBCs) with no xanthochromia excluded a diagnosis of aneurysmal SAH (sensitivity 100% (95% CI 74.7 to 100), specificity 91.2% (95% CI 88.6 to 93.3)) in patients who had previously undergone CT.^41^ Heiser *et al* assessed the same RBC cut-off, reporting 81.6% sensitivity (95% CI 68.0 to 91.2) and 97.3% specificity (95% CI 95.7 to 98.4); the incidence of traumatic LP was 24.4%.^37^ These results are not directly comparable to those reported by Perry *et al*,^41^ as this population was not prescreened with CT.

Finally, Valle Alonso *et al* assessed 74 patients who underwent LP (method of analysis not specified) following negative CT <6 hours.^30^ LP was positive in one patient and inconclusive in two; further imaging ruled out bleeding in all three patients. Seven patients experienced postpuncture headache, two of whom were admitted for pain control.

### CT angiography

Two small studies assessed CTA after normal CT/LP; no cases of SAH were identified, although other vascular abnormalities (including incidental aneurysms, cerebral venous thrombosis and reversible vasoconstriction syndrome) were identified.^44 45^

### History and examination

Three studies explored the use of historical and emergent clinical factors as predictors of SAH.^2 46 47^ Two studies investigated the adequacy of assessment for SAH and one study assessed neurological examination for neck stiffness as a predictor of SAH. Using physicians’ clinical suspicion had a sensitivity 93% and specificity of 49%.^46^ Presence of individual clinical factors (age >65 years, temperature >38**°**C, systolic BP >160 mm Hg, neck stiffness) were poor predictors of secondary headache (sensitivity 37.8%, specificity 82.1%).^2^ Presence of neck stiffness was more strongly predictive of SAH in patients who had other high-risk clinical characteristics (eg, age ≥40 years, vomiting, transient loss of consciousness).^47^ Recording of history in medical records was poor.^2 46 47^

## Discussion

In summary, the Ottawa SAH Rule does little to aid clinical decision making for patients with sudden onset severe headache. The FPR was high, such that 76% of SAH-negative patients would undergo further investigation with CT and/or LP with no diagnostic value with regard to SAH, resulting in greater healthcare resource use and higher rates of adverse events related to LP and CT radiation exposure. Evidence on use of the rule in patient subgroups by time to headache peak is lacking but could be informative for clinical practice given the importance of headache incipiency.

LP (with spectrophotometric CSF analysis) following negative CT was highly sensitive, although there was a 4.8% FPR. Spectrophotometry-based CSF analysis appeared to have a higher sensitivity but lower specificity than visual inspection for xanthochromia. Two studies reported rates of LP-related complications resulting in a return to the ED or hospitalisation (5%–10%). In view of the reduced sensitivity of CT >6 hours after headache onset, LP may be beneficial in these patients where a clinical suspicion of SAH remains. The CT–LP pathway also identified other significant pathologies, such as intracerebral haemorrhage, brain tumour and meningitis, meaning that its value could extend beyond the identification of SAH.

Non-contrast CT <6 hours from headache onset, with CT scans assessed by a neuroradiologist or radiologist who routinely interprets head CT images, is highly accurate for identifying SAH, and results in a very low post-test probability of SAH. This means that very large numbers of patients (estimated at 658) would have to undergo further testing to yield an additional case of SAH.

However, the relatively high rate of false positive LP results (4.8% using spectrophotometry) is likely to lead to yet more testing downstream with the potential for diagnosing incidental aneurysms, leading to difficult decisions about invasive procedures. A 2016 survey of UK clinicians reported a higher risk tolerance for missed SAH diagnoses among emergency clinicians than neurospecialists, with the former accepting over 2.5 times the risk of a missed SAH (2.8% vs 1.1%; p=0.03), and the latter more likely to advocate routine LP following a negative CT result (74% vs 39%; p=0.01).^4^ Emergency clinicians were also more inclined to omit LP if CT had been conducted within 6 hours of headache onset (35% vs 3%; p=0.002).

Draft guidelines by the National Institute for Health and Care Excellence (publication delayed due to COVID-19) recommend that when there is no evidence of SAH on CT images taken <6 hours from symptom onset, LP should not be routinely offered, and alternative diagnoses should instead be considered.^48^ However, we consider that in smaller centres without access to specialist neuroradiology expertise, or radiologists who routinely interpret head CTs, the accuracy of early CT may be reduced; studies included in our meta-analyses benefited from neuroradiology expertise. Introduction of universal access to expert interpretation of CT images could improve SAH-related patient outcomes through optimised targeting of further investigations while increasing efficiency of resource allocation. This may be achieved through widened neuro-specific training and teleradiology using other centres with relevant expertise. While interpretation of CT images using diagnostic deep learning algorithms (artificial intelligence) has the potential to improve consistency across centres, this has yet to be reliably demonstrated in high-quality studies.^49^

The prevalence of SAH was higher in patients who received CT <6 hours from headache onset than in the wider population of patients presenting to the ED with sudden onset severe headache (10.8% vs 7.0%). It is unclear whether this difference in pre-test probability can be assumed to exist at the point of patient assessment in the ED. Instead, triage based on severity of symptoms may have reduced wait time for CT, equally, symptom severity associated with true SAH could drive earlier presentation.

A limitation of this review was the substantial heterogeneity in the study methods and population characteristics of the included studies. The evidence base included too few patients, given the rarity of SAH events, missed diagnoses and alternative non-SAH pathologies. This led to heterogeneity in the results of some meta-analyses, and potentially meant uncertainty was underestimated in others.

There was a lack of research evidence on the small subgroup of patients who present to hospital several days after headache onset. Diagnosis of SAH in such patients is particularly challenging and there is a lack of guidance and consistency in how these patients are assessed.

## Conclusions

The Ottawa SAH Rule rules out further investigation in only a small proportion of patients; its introduction into practice could result in substantially increased rates of unnecessary investigation. Assuming the availability of neuroradiology expertise, early head CT (<6 hours) appears to be sufficient to rule out SAH in patients with sudden onset severe headache in the vast majority of patients. CT undertaken >6 hours from headache onset is much less sensitive, therefore, LP is more likely to be beneficial, where a clinical suspicion of SAH remains. Risk tolerance of the patient and the physician, the expertise of the CT reader and consequences of additional investigations must all be considered.

## Data Availability

All data relevant to the study are included in the article or uploaded as supplementary information. Not applicable.
