# Supplementary material for: Management of patients presenting to the emergency department with sudden onset severe headache: systematic review of diagnostic accuracy studies
Source: Emerg Med J. 2022 Mar 31;39(11):818–25. doi: 10.1136/emermed-2021-211900 (PMC9613855; doi:10.1136/emermed-2021-211900)
Supplement: Supplementary data [file emermed-2021-211900supp001.pdf]

Supplementary file 1 Database search strategies

MEDLINE ALL
(includes: Epub Ahead of Print, In-Process & Other Non-Indexed Citations, Ovid MEDLINE Daily and Ovid MEDLINE)
via Ovid http://ovidsp.ovid.com/
1946 to February 07, 2020
Searched on: 10th February 2020
Records retrieved: 5141

1 Headache Disorders, Primary/ (771)
2 Headache/ (27331)
3 Vascular Headaches/ (1301)
4 Headache Disorders, Secondary/ (604)
5 Headache Disorders/ (2300)
6 (headache$ or head ache$).ti,ab. (81511)
7 LASH.ti,ab. (377)
8 (thunderclap$ or thunder clap$).ti,ab. (483)
9 (cephalalgi$ or cephalgi$).ti,ab. (1088)
10 (cranial adj2 pain$).ti,ab. (180)
11 (hemicrania or cephalea or cranialgia).ti,ab. (1015)
12 or/1-11 (91905)
13 Subarachnoid Hemorrhage/ (20706)
14 (Subarachnoid$ adj2 hemorr?ag$).ti,ab. (20324)
15 (Subarachnoid$ adj2 haemorr?ag$).ti,ab. (4429)
16 (Subarachnoid$ adj2 (bleed$ or blood)).ti,ab. (796)
17 (arachnoid$ adj2 (haemorr?ag$ or hemorr?ag$ or bleed$ or blood)).ti,ab. (210)
18 (SAH or SAHs).ti,ab. (10673)
19 or/13-18 (32469)
20 12 and 19 (2380)
21 Emergencies/ (39849)
22 Emergency Service, Hospital/ (66056)
23 exp Emergency Medical Services/ (136120)
24 Triage/ (11201)
25 ((emergency or emergencies or casualty) adj3 (room$ or department$ or service$ or unit$ or ward$ or centre$ or center$ or hospital$ or setting$ or clinic or clinics or care or healthcare or medical)).ti,ab. (149559)
26 triage$.ti,ab. (17352)
27 (accident$ adj2 (emergency or emergencies)).ti,ab. (4771)
28 21 or 22 or 23 or 24 or 25 or 26 or 27 (259901)
29 12 and 28 (3064)
30 20 or 29 (5154)
31 exp animals/ not humans/ (4671979)
32 30 not 31 (5141)

EMBASE
via Ovid http://ovidsp.ovid.com/
1974 to 2020 February 07
Searched on: 10th February 2020
Records retrieved: 13950

1 "headache and facial pain"/ (1630)
2 secondary headache/ (1161)
3 headache/ (208066)
4 vascular headache/ (574)
5 thunderclap headache/ (788)
6 exertional headache/ (102)
7 stabbing headache/ (226)
8 exp tension headache/ (7654)
9 (headache$ or head ache$).ti,ab. (129375)
10 LASH.ti,ab. (554)
11 (thunderclap$ or thunder clap$).ti,ab. (838)
12 (cephalalgi$ or cephalgi$).ti,ab. (1810)
13 (cranial adj2 pain$).ti,ab. (250)
14 (hemicrania or cephalea or cranialgia).ti,ab. (1411)
15 or/1-14 (253856)
16 subarachnoid hemorrhage/ (42006)
17 (Subarachnoid$ adj2 hemorr?ag$).ti,ab. (26796)
18 (Subarachnoid$ adj2 haemorr?ag$).ti,ab. (6005)
19 (Subarachnoid$ adj2 (bleed$ or blood)).ti,ab. (1037)
20 (arachnoid$ adj2 (haemorr?ag$ or hemorr?ag$ or bleed$ or blood)).ti,ab. (403)
21 (SAH or SAHs).ti,ab. (15683)
22 16 or 17 or 18 or 19 or 20 or 21 (50197)
23 15 and 22 (5697)
24 Emergency/ (52475)
25 Emergency health service/ (94019)
26 Hospital emergency service/ (4243)
27 Emergency ward/ (138545)
28 Emergency care/ (43804)
29 Emergency patient/ (3295)
30 ((emergency or emergencies or casualty) adj3 (room$ or department$ or service$ or unit$ or ward$ or centre$ or center$ or hospital$ or setting$ or clinic or clinics or care or healthcare or medical)).ti,ab. (227136)
31 triage$.ti,ab. (27449)
32 (accident$ adj2 (emergency or emergencies)).ti,ab. (6037)
33 24 or 25 or 26 or 27 or 28 or 29 or 30 or 31 or 32 (380671)
34 15 and 33 (8994)
35 23 or 34 (13959)
36 (rat or rats or mouse or mice).ti. (1445465)
37 35 not 36 (13950)

Cochrane Central Register of Controlled Trials (CENTRAL)
via Wiley http://onlinelibrary.wiley.com/
Issue 2 of 12, February 2020
Searched on: 10th February 2020
Records retrieved: 581

The strategy below was used to search both CENTRAL and CDSR.

#1 MeSH descriptor: [Headache Disorders] this term only 135
#2 MeSH descriptor: [Headache] this term only 2318
#3 MeSH descriptor: [Vascular Headaches] this term only 40
#4 MeSH descriptor: [Headache Disorders, Secondary] this term only 55
#5 MeSH descriptor: [Headache Disorders, Primary] this term only 17
#6 (headache* or head next ache*):ti,ab,kw 31430
#7 LASH:ti,ab,kw 90
#8 (thunderclap* or thunder next clap*):ti,ab,kw 4
#9 (cephalalgi* or cephalgi*):ti,ab,kw 76
#10 (cranial near/2 pain*):ti,ab,kw 13
#11 (hemicrania or cephalea or cranialgia):ti,ab,kw 46
#12 #1 or #2 or #3 or #4 or #5 or #6 or #7 or #8 or #9 or #10 or #11 31574
#13 MeSH descriptor: [Subarachnoid Hemorrhage] this term only 579
#14 (Subarachnoid* near/2 hemorr?ag*):ti,ab,kw 1819
#15 (Subarachnoid* near/2 haemorr?ag*):ti,ab,kw 470
#16 (Subarachnoid* near/2 (bleed* or blood)):ti,ab,kw 55
#17 (arachnoid* near/2 (haemorr?ag* or hemorr?ag* or bleed* or blood)):ti,ab,kw 32
#18 (SAH or SAHs):ti,ab,kw 1011
#19 #13 or #14 or #15 or #16 or #17 or #18 2336
#20 #12 and #19 90
#21 MeSH descriptor: [Emergencies] this term only 1318
#22 MeSH descriptor: [Emergency Service, Hospital] this term only 2111
#23 MeSH descriptor: [Emergency Medical Services] explode all trees 3734
#24 MeSH descriptor: [Triage] this term only 285
#25 ((emergency or emergencies or casualty) near/3 (room* or department* or service* or unit* or ward* or centre* or center* or hospital* or setting* or clinic or clinics or care or healthcare or medical)):ti,ab,kw 18260
#26 triage*:ti,ab,kw 1717
#27 (accident* near/2 (emergency or emergencies)):ti,ab,kw 355
#28 #21 or #22 or #23 or #24 or #25 or #26 or #27 20016
#29 #12 and #28 509
#30 #20 or #29 592
#31 #20 or #29 in Trials 581
#32 #20 or #29 in Cochrane Reviews, Cochrane Protocols 11

Cochrane Database of Systematic Reviews (CDSR)
via Wiley http://onlinelibrary.wiley.com/
Issue 2 of 12, February 2020
Searched on: 10th February 2020
Records retrieved: 11

See above under CENTRAL for search strategy used.

Science Citation Index
via Web of Science, Clarivate Analytics https://clarivate.com/
1900 – 7th February 2020
Searched on: 10th February 2020
Records retrieved: 3758

21 3,758 #19 not #20
20 1,684,685 TI=(rat or rats or mouse or mice)
19 3,765 #18 OR #13
18 2,204 #17 AND #6
17 142,684#16 OR #15 OR #14
16 3,670 TS=(accident* NEAR/2 emergenc*)
15 16,817 TS=triage*
14 131,101TS=((emergency or emergencies or casualty) NEAR/3 (room* or department* or service* or unit* or ward* or centre* or center* or hospital* or setting* or clinic or clinics or care or healthcare or medical))
13 1,787 #12 AND #6
12 32,430 #11 OR #10 OR #9 OR #8 OR #7
11 9,617 TS=(SAH or SAHs)
10 416 TS=(arachnoid* NEAR/2 (haemorr$ag* or hemorr$ag* or bleed* or blood))
9 856 TS=(Subarachnoid* NEAR/2 (bleed* or blood))
8 3,745 TS=(Subarachnoid* NEAR/2 haemorr$ag*)
7 26,313 TS=(Subarachnoid* NEAR/2 hemorr$ag*)
6 73,503 #5 OR #4 OR #3 OR #2 OR #1
5 1,111 TS=(hemicrania or cephalea or cranialgia)
4 219 TS=(cranial NEAR/2 pain*)
3 1,139 TS=(cephalalgi* or cephalgi*)
2 584 TS=(thunderclap* or "thunder clap*")
1 72,669 TS=(headache* or "head ache*" or LASH)

Database of Abstracts of Reviews of Effects (DARE)
via http://www.crd.york.ac.uk/CRDWeb/
Inception – 31st March 2015
Searched on: 10th February 2020
Records retrieved: 19

The strategy below was used to search all three of the CRD databases - DARE, the HTA database and NHS EED.

1 MeSH DESCRIPTOR Headache Disorders, Primary 1
2 MeSH DESCRIPTOR Headache 81
3 MeSH DESCRIPTOR Vascular Headaches 0
4 MeSH DESCRIPTOR Headache Disorders, Secondary 2
5 MeSH DESCRIPTOR Headache Disorders 21
6 (headache* or "head ache" or "head aches") 806
7 (thunderclap* or thunder clap*) 1
8 (cephalalgi* or cephalgi*) 36
9 (cranial NEAR2 pain*) 0
10 (pain* NEAR2 cranial) 0
11 (hemicrania or cephalea or cranialgia) 2
12 (LASH) 3
13 #1 OR #2 OR #3 OR #4 OR #5 OR #6 OR #7 OR #8 OR #9 OR #10 OR #11 OR #12 819
14 MeSH DESCRIPTOR Subarachnoid Hemorrhage 96
15 (Subarachnoid* NEAR2 (haemorrhag* or hemorrhag* or haemorrag* or hemorrag*)) 158
16 ((haemorrhag* or hemorrhag* or haemorrag* or hemorrag*) NEAR2 subarachnoid*) 5
17 (Subarachnoid* NEAR2 (bleed* or blood)) 0
18 ((bleed* or blood) NEAR2 Subarachnoid*) 1
19 (arachnoid* NEAR2 (haemorrhag* or hemorrhag* or haemorrag* or hemorrag* or bleed* or blood)) 6
20 ((haemorrhag* or hemorrhag* or haemorrag* or hemorrag* or bleed* or blood) NEAR2 arachnoid*) 0
21 (SAH or SAHs) 44
22 #14 OR #15 OR #16 OR #17 OR #18 OR #19 OR #20 OR #21 168
23 #13 AND #22 4
24 MeSH DESCRIPTOR Emergencies 86
25 MeSH DESCRIPTOR Emergency Service, Hospital 442
26 MeSH DESCRIPTOR Emergency Medical Services EXPLODE ALL TREES 825
27 MeSH DESCRIPTOR Triage 111
28 ((emergency or emergencies or casualty) NEAR3 (room* or department* or service* or unit* or ward* or centre* or center* or hospital* or setting* or clinic or clinics or care or healthcare or medical)) 1927
29 ((room* or department* or service* or unit* or ward* or centre* or center* or hospital* or setting* or clinic or clinics or care or healthcare or medical) NEAR3 (emergency or emergencies or casualty)) 727
30 (triage*) 258
31 (accident* NEAR2 (emergency or emergencies)) 121
32 ((emergency or emergencies) NEAR2 accident*) 2
33 #24 OR #25 OR #26 OR #27 OR #28 OR #29 OR #30 OR #31 OR #32 2279
34 #13 AND #33 44
35 #23 OR #34 46

Health Technology Assessment (HTA) database
via http://www.crd.york.ac.uk/CRDWeb/
Inception – 31st March 2018
Searched on: 10th February 2020
Records retrieved: 1

See above under DARE for search strategy used.

NHS Economic Evaluations Database (NHS EED)
via http://www.crd.york.ac.uk/CRDWeb/
Inception – 31st March 2015
Searched on: 10th February 2020
Records retrieved: 26

See above under DARE for search strategy used.

EconLit
via Ovid http://ovidsp.ovid.com/
1886 to January 30, 2020
Searched on: 10th February 2020
Records retrieved: 1

1 (headache$ or head ache$).ti,ab. (57)
2 LASH.ti,ab. (9)
3 (thunderclap$ or thunder clap$).ti,ab. (0)
4 (cephalalgi$ or cephalgi$).ti,ab. (0)
5 (cranial adj2 pain$).ti,ab. (0)
6 (hemicrania or cephalea or cranialgia).ti,ab. (0)
7 or/1-6 (66)
8 (Subarachnoid$ adj2 hemorr?ag$).ti,ab. (1)
9 (Subarachnoid$ adj2 haemorr?ag$).ti,ab. (1)
10 (Subarachnoid$ adj2 (bleed$ or blood)).ti,ab. (0)
11 (arachnoid$ adj2 (haemorr?ag$ or hemorr?ag$ or bleed$ or blood)).ti,ab. (0)
12 (SAH or SAHs).ti,ab. (52)
13 8 or 9 or 10 or 11 or 12 (53)
14 7 and 13 (0)
15 ((emergency or emergencies or casualty) adj3 (room$ or department$ or service$ or unit$ or ward$ or centre$ or center$ or hospital$ or setting$ or clinic or clinics or care or healthcare or medical)).ti,ab. (667)
16 triage$.ti,ab. (78)
17 (accident$ adj2 (emergency or emergencies)).ti,ab. (22)
18 15 or 16 or 17 (732)
19 7 and 18 (1)
20 14 or 19 (1)

On-going, unpublished or grey literature searches

ClinicalTrials.gov
https://clinicaltrials.gov/
Searched on: 11th February 2020
Records retrieved: 139

1. 20 Studies found for: headache AND (subarachnoid haemorrhage OR subarachnoid haemorrhage OR sub-arachnoid haemorrhage OR sub-arachnoid hemorrhage)

2. 1 Study found for: thunderclap AND (subarachnoid haemorrhage OR subarachnoid haemorrhage OR sub-arachnoid haemorrhage OR sub-arachnoid hemorrhage)

3. 2 Studies found for: headache AND (arachnoid haemorrhage OR arachnoid hemorrhage)

4. No Studies found for: thunderclap AND (arachnoid haemorrhage OR arachnoid hemorrhage)

5. 116 Studies found for: headache AND (emergency OR casualty OR triage)

6. No Studies found for: thunderclap AND (emergency OR casualty OR triage)

WHO International Clinical Trials Registry Platform
http://www.who.int/ictrp/search/en/
Searched on: 11th February 2020
Records retrieved: 84

Basic search interface used.

1. 13 records for 13 trials found for: headache AND subarachnoid

2. 1 trial found for: headache AND sub-arachnoid

3. 1 trial found for: headache AND arachnoid

4. No results were found for: thunderclap OR thunder clap

5. 68 records for 68 trials found for: headache AND emergenc*

6. No results were found for: headache AND casualty

7. 1 trial found for: headache AND triag*

EU Clinical Trials Register
https://www.clinicaltrialsregister.eu/ctr-search/search
Searched on: 11th February 2020
Records retrieved: 16

1. 3 result(s) found for: headache* AND (subarachnoid* haemorrhag* OR subarachnoid* haemorrhag* OR sub-arachnoid* haemorrhage* OR sub-arachnoid* hemorrhage*)

2. thunderclap OR "thunder clap" – 0 results

3. 2 result(s) found for: headache* AND (arachnoid* haemorrhag* OR arachnoid* hemorrhag*)

4. 11 result(s) found for: headache* AND (emergenc* OR casualty OR triag*)

Conference Proceedings Citation Index: Science
via Web of Science, Clarivate Analytics https://clarivate.com/
1990 – 7th February 2020
Searched on: 10th February 2020
Records retrieved: 251

19 251 #18 OR #13
18 193 #17 AND #6
17 18,714 #16 OR #15 OR #14
16 511 TS=(accident* NEAR/2 emergenc*)
15 2,397 TS=triage*
14 16,654 TS=((emergency or emergencies or casualty) NEAR/3 (room* or department* or service* or unit* or ward* or centre* or center* or hospital* or setting* or clinic or clinics or care or healthcare or medical))
13 70 #12 AND #6
12 3,066 #11 OR #10 OR #9 OR #8 OR #7
11 990 TS=(SAH or SAHs)
10 22 TS=(arachnoid* NEAR/2 (haemorr$ag* or hemorr$ag* or bleed* or blood))
9 44 TS=(Subarachnoid* NEAR/2 (bleed* or blood))
8 388 TS=(Subarachnoid* NEAR/2 haemorr$ag*)
7 2,317 TS=(Subarachnoid* NEAR/2 hemorr$ag*)
6 7,771 #5 OR #4 OR #3 OR #2 OR #1
5 94 TS=(hemicrania or cephalea or cranialgia)
4 8 TS=(cranial NEAR/2 pain*)
3 92 TS=(cephalalgi* or cephalgi*)
2 56 TS=(thunderclap* or "thunder clap*")
1 7,652 TS=(headache* or "head ache*" or LASH)

PROSPERO
http://www.crd.york.ac.uk/PROSPERO/
Searched on: 11th February 2020
Records retrieved: 60

#1 MeSH DESCRIPTOR Headache Disorders, Primary 3
#2 MeSH DESCRIPTOR Headache 62
#3 MeSH DESCRIPTOR Vascular Headaches 0
#4 MeSH DESCRIPTOR Headache Disorders, Secondary 4
#5 MeSH DESCRIPTOR Headache Disorders 18
#6 headache* or (head adj1 ache*) 865
#7 headache* or "head ache" or "head aches" 865
#8 LASH 7
#9 thunderclap* or (thunder adj1 clap*) 1
#10 cephalalgi* or cephalgi* 31
#11 cranial adj2 pain* 2
#12 hemicrania or cephalea or cranialgia 7
#13 #1 OR #2 OR #3 OR #4 OR #5 OR #6 OR #8 OR #9 OR #10 OR #11 OR #12 876
#14 MeSH DESCRIPTOR Subarachnoid Hemorrhage 62
#15 Subarachnoid* adj2 (hemorrhag* or hemorrag* or haemorrhag* or haemorrag*) 221
#16 Subarachnoid* adj2 (bleed* or blood) 14
#17 arachnoid* adj2 (haemorrhag* or haemorrag* or hemorrhag* or hemorrag* or bleed* or blood) 4
#18 SAH or SAHs 95
#19 #14 OR #15 OR #16 OR #17 OR #18 247
#20 #13 AND #19 8
#21 MeSH DESCRIPTOR Emergencies 81
#22 MeSH DESCRIPTOR Emergency Service, Hospital 303
#23 MeSH DESCRIPTOR Emergency Medical Services EXPLODE ALL TREES 493
#24 MeSH DESCRIPTOR Triage 50
#25 (emergency or emergencies or casualty) adj3 (room* or department* or service* or unit* or ward* or centre* or center* or hospital* or setting* or clinic or clinics or care or healthcare or medical) 2230
#26 triage* 230
#27 accident* adj2 (emergency or emergencies) 184
#28 #21 OR #22 OR #23 OR #24 OR #25 OR #26 OR #27 2477
#29 #28 AND #13 53
#30 #20 OR #29 60

ECRI Guidelines Trust
https://guidelines.ecri.org/
Searched on: 17th February 2020
Records retrieved: 5

1. headache OR thunderclap OR “thunder clap” – 39 results – filtered to diagnosis – 16 results browsed for relevance – 5 potentially relevant.

Clinical Knowledge Summaries
https://cks.nice.org.uk/
Searched on: 17th February 2020
Records retrieved: 4

Browsed topic list for headache – 4 relevant records found.

NHS Evidence
https://www.evidence.nhs.uk/
Searched on: 17th February 2020
Records retrieved: 69

The following search strings were entered into the search box with the inbuilt guidance filters box checked to limit results to guidelines.

1. headache* AND "subarachnoid haemorrhage" – filtered to guidance - 26 results

2. headache* AND "subarachnoid hemorrhage" – filtered to guidance – 19 results

3. (intitle: headache*) AND emergenc* - filtered to guidance – 24 results

Trip
https://www.tripdatabase.com/
Searched on: 25th February 2020
Records retrieved: 17

1. (title:headache) AND ("subarachnoid haemorrhage" OR "subarachnoid hemorrhage") – filtered to guidance – 7 results

2. (title:headache) AND emergency – filtered to guidance – 10 results
