# Supplementary material for: Management of patients presenting to the emergency department with sudden onset severe headache: systematic review of diagnostic accuracy studies
Source: Emerg Med J. 2022 Mar 31;39(11):818–25. doi: 10.1136/emermed-2021-211900 (PMC9613855; doi:10.1136/emermed-2021-211900)
Supplement: Supplementary data [file emermed-2021-211900supp002.pdf]

Supplementary File 2 Characteristics and results of studies included in the systematic review

**Study details**	**Patient characteristics**	**Intervention**	**Reference standard**	**Results**	**Risk of bias**
**Canadian clinical decision rules (Rule 1, 2 and 3 and the Ottawa SAH Rule)**					
Perry, 2010[25]<br><br>Prospective cohort study<br><br>Emergency Departments at six university affiliated tertiary care teaching hospitals, Canada<br><br>*Also reported in CT scan section*	1999 non-traumatic, alert, neurologically intact (GCS 15) headache patients (peaking within 1 hour) or syncope associated with headache. An additional 1050 potentially eligible patients were identified who were not enrolled 'missed eligible patients'.<br><br>Patient recruitment: November 2000 – November 2005 (patient overlap with Perry, 2011[43]).	Third generation CT (results verified by either a neuroradiologist or general radiologist who routinely interprets head CT).<br><br>Identification of high risk clinical characteristics for SAH in order to develop clinical decision rules based on variables collected on history or examination.<br><br>Rule 1: age >40; complaint of neck pain or stiffness; witnessed loss of consciousness; onset with exertion.<br><br>Rule 2: arrival by ambulance; age >45; vomiting at least	CT, LP (xanthochromia on visual inspection or >5x10^6/L RBCs in the final tube of CSF with aneurysm or arteriovenous malformation seen on angiography) and clinical follow-up (telephone follow-up at 1 month and 6 months and medical record review).	**Diagnostic accuracy results**<br><br>**CT (SAH):**<br><br>Sensitivity: 93.1% (calculated by CRD)<br><br>Specificity: 100% (calculated by CRD)<br><br>Positive predictive value: 100% (calculated by CRD)<br><br>Negative predictive value: 99.4% (calculated by CRD)<br><br>Overall accuracy: 99.4% (calculated by CRD)<br><br>Prevalence: 6.5%<br><br>**Clinical decision rules (SAH):**<br><br>Retrospective sensitivity: Rule 1-3: 100% (95% CI 97.1 to 100)<br><br>Specificity: Rule 1: 28.4% (95% CI 26.4 to 30.4); Rule 2: 36.5% (95% CI	Patient selection: Unclear<br><br>Index test: Low<br><br>Reference standard: Low<br><br>Flow/timing: Low

		once; diastolic BP >100 mm Hg.<br><br>Rule 3: arrival by ambulance; systolic BP >160 mm Hg; complaint of neck pain or stiffness; age 45-55.		34.4 to 38.8); Rule 3: 38.8% (95% CI 36.7 to 41.1).<br><br>**Diagnostic tests performed**<br><br>1606 (80.3%) patients had a CT scan and 905 (45.3%) had LP; 854 (42.7%) had CT scan and LP. 8.4% patients had a CT angiogram. Use of any one of the rules assessed would have lowered rates of investigation (CT, LP or both) from 82.9% to between 63.7-73.5%.<br><br>**Other significant diagnoses**<br><br>48 patients had other serious conditions diagnosed on CT or LP, such as transient ischaemic attack/acute ischaemic stroke, other type of haemorrhagic stroke, bacterial meningitis, hypertensive emergency or cerebral neoplasm.	
Perry, 2013[26]<br><br>Prospective cohort study	2131 non-traumatic, neurologically intact (GCS 15) headache patients (peaking within 1 hour).	3 clinical decision rules and development of the Ottawa SAH Rule.<br><br>Rule 1: age >40; complaint of neck pain or stiffness;	CT, LP (xanthochromia on visual inspection or >1x10^6/L RBCs in the final tube of CSF with aneurysm or arteriovenous malformation seen on	**Diagnostic accuracy results**<br><br>**Rule 1 (SAH):**<br><br>Sensitivity: 98.5% (95% CI 94.6 to 99.6)	Patient selection: Low<br><br>Index test: Low<br><br>Reference standard: Low

Emergency Departments at ten university hospitals, Canada	Patient recruitment: April 2006 – July 2010 (appears to be patient overlap with Perry, 2011[43]).	witnessed loss of consciousness; onset with exertion.<br><br>Rule 2: arrival by ambulance; age >45; vomiting at least once; diastolic BP >100 mm Hg.<br><br>Rule 3: arrival by ambulance; systolic BP >160 mm Hg; complaint of neck pain or stiffness; age 45-55.<br><br>Ottawa SAH Rule: age >40; complaint of neck pain or stiffness; witnessed loss of consciousness; onset with exertion; thunderclap headache (instantly peaking pain); limited neck flexion on examination.	angiography) and clinical follow-up (telephone follow-up at 1 month and 6 months and medical record review).	Specificity: 27.6% (95% CI 25.7 to 29.6)<br><br>**Rule 2 (SAH):**<br><br>Sensitivity: 95.5% (95% CI 90.4 to 97.9)<br><br>Specificity: 30.6% (95% CI 28.6 to 32.6)<br><br>**Rule 3 (SAH):**<br><br>Sensitivity: 97.0% (95% CI 92.5 to 98.8)<br><br>Specificity: 35.6% (95% CI 33.6 to 37.7)<br><br>**Ottawa SAH Rule (SAH):**<br><br>Sensitivity: 100% (95% CI 97.2 to 100)<br><br>Specificity: 15.3% (95% CI 13.8 to 16.9)<br><br>Positive predictive value: 7.2% (calculated by CRD)	Flow/timing: Low

				Negative predictive value: 100% (calculated by CRD)<br><br>Overall accuracy: 20.5% (calculated by CRD)<br><br>Prevalence: 6.2%<br><br>**Physician survey**<br><br>Physicians were ‘uncomfortable’ or ‘very uncomfortable’ using rule 1 in 18.2% patients, rule 2 in 23.7% patients and rule 3 in 23.6% patients. Physicians misinterpreted the clinical decision rule as not requiring investigation in 4.7% patients using rule 1, 6.0% using rule 2 and 4.6% using rule 3 – the most frequently misinterpreted variables were neck pain and stiffness for rules 1 and 3 and arrival by ambulance for rule 2.<br><br>**Diagnostic tests performed**<br><br>1767 (82.9%) patients had a CT scan and 833 (39.1%) had LP. 15.1% patients had a CT angiogram. 84.3% patients had CT, LP or both; use of rule 1 would have decreased this rate	

				to 74.0%, rule 2 to 71.0% and rule 3 to 66.4%. The Ottawa SAH Rule would have slightly increased the investigation rate to 85.7%.	
Matloob, 2013[24]<br><br>Retrospective cohort study, UK<br><br>Emergency Department at one teaching hospital, UK	112 non-traumatic, alert, neurologically intact (GCS 15) headache patients (peaking within 1 hour).<br><br>Patient recruitment: August 2011 – October 2011.	UK validation of 3 Canadian clinical decision rules.<br><br>Rule 1: age >40; complaint of neck pain or stiffness; witnessed loss of consciousness; onset with exertion.<br><br>Rule 2: arrival by ambulance; age >45; vomiting at least once; diastolic BP >100 mm Hg.<br><br>Rule 3: arrival by ambulance; systolic BP >160 mm Hg; complaint of neck pain or stiffness; age 44-55.<br><br>Comparator: Current UK practice (defined as clinical	Diagnosis on discharge. SAH was defined using CT and LP (xanthochromia). In patients not fully investigated the authors searched for admission to regional neurosurgical centre within 6 months of discharge.	**Diagnostic accuracy results**<br><br>**Rule 1 (SAH):**<br><br>Sensitivity: 100% (95% CI 40 to 100)<br><br>Specificity: 43% (95% CI 33 to 52)<br><br>Positive predictive value: 6.1% (calculated by CRD)<br><br>Negative predictive value: 100% (95% CI 90 to 100)<br><br>Overall accuracy: 44.6% (calculated by CRD)<br><br>**Rule 2 (SAH):**<br><br>Sensitivity: 100% (95% CI 40 to 100)<br><br>Specificity: 27% (95% CI 19 to 36)<br><br>Positive predictive value: 4.8% (calculated by CRD)	Patient selection: Low<br><br>Index test: Unclear<br><br>Reference standard: Unclear<br><br>Flow/timing: High

		assessment without the use of a formal decision rule).		Negative predictive value: 100% (95% CI 85 to 100)<br><br>Overall accuracy: 29.5% (calculated by CRD)<br><br>**Rule 3 (SAH):**<br><br>Sensitivity: 100% (95% CI 40 to 100)<br><br>Specificity: 37% (95% CI 28 to 47)<br><br>Positive predictive value: 5.6% (calculated by CRD)<br><br>Negative predictive value: 100% (95% CI 89 to 100)<br><br>Overall accuracy: 39.3% (calculated by CRD)<br><br>**Current UK practice (SAH):**<br><br>Sensitivity: 100% (95% CI 40 to 100)<br><br>Specificity: 66% (95% CI 56 to 74)<br><br>Positive predictive value: 9.8% (calculated by CRD)	

				Negative predictive value: 100% (95% CI 94 to 100)<br><br>Overall accuracy: 67.0% (calculated by CRD)<br><br>Prevalence: 3.6%<br><br>**Diagnostic tests performed**<br><br>41 (36.6%) patients had a CT scan and 9 (8.0%) had LP (after –ve CT). The investigation rate of 36.6% would have increased with the use of the Canadian decision rules (59%, 74% and 64% for rules 1-3 respectively).	
MacDonald, 2012[23]<br><br>Retrospective cohort study<br><br>Emergency department at one District General Hospital, UK	280 neurologically intact, acute headache patients who had head CT.<br><br>Patient recruitment: 2 year period.	Perry's three decision rules to aid investigation of suspected SAH.	CT. LP results were searched for patients with suspected SAH but no evidence on CT.	**Diagnostic accuracy results (SAH and other significant diagnoses)**<br><br>8/280 (2.9%) patients had SAH. None would have been missed using the clinical decision rules suggested by Perry *et al.* However, there were nine cases of other significant pathologies such as intra-parenchymal bleeds, tumours and infarction that would have been missed by employing the rules.	Patient selection: Unclear<br><br>Index test: Unclear<br><br>Reference standard: Low<br><br>Flow/timing: Unclear<br><br>(limited reporting, as only a conference

					abstract was available)
Kelly, 2014[22]<br><br>Retrospective cohort study<br><br>Emergency Departments at two teaching hospitals, Australia	59 non-traumatic neurologically intact (GCS 15) sudden onset headache patients with confirmed SAH (all were confirmed with CT).<br><br>Patient recruitment: 2000 – 2011.	3 Canadian clinical decision rules.<br><br>Rule 1: age >40; complaint of neck pain or stiffness; witnessed loss of consciousness; onset with exertion.<br><br>Rule 2: arrival by ambulance; age >45; vomiting at least once; diastolic BP >100 mm Hg.<br><br>Rule 3: arrival by ambulance; systolic BP >160 mm Hg; complaint of neck pain or stiffness; age 45-55.	CT, CT angiography, conventional angiography, MRI, or LP supported by specialist neurosurgical opinion.	**Diagnostic accuracy results**<br><br>**Rule 1 (SAH):**<br><br>Sensitivity: 96.6% (95% CI 88.5 to 99.1); 2 cases missed.<br><br>**Rule 2 (SAH):**<br><br>Sensitivity: 100% (95% CI 93.9 to 100)<br><br>**Rule 3 (SAH):**<br><br>Sensitivity: 89.8% (95% CI 79.5 to 95.3); 6 cases missed.<br><br>The addition of vomiting to rule 1 and 3 increased sensitivity to 100%.	Patient selection: Low<br><br>Index test: High<br><br>Reference standard: Low<br><br>Flow/timing: High
Yiangou, 2017[27]<br><br>Retrospective cohort study	162 fully alert, neurologically intact patients presenting with acute headache.	Four Canadian SAH decision rules: Rule 1, Rule 2, Rule 3 and the Ottawa SAH Rule (full results only presented for the Ottawa SAH Rule).	Final diagnosis (CT, LP and re-admission with SAH).	**Diagnostic accuracy results**<br><br>**Ottawa SAH Rule:**<br><br>Sensitivity: 100% (95% CI 31.0 to 100)	Patient selection: Unclear<br><br>Index test: Unclear<br><br>Reference standard: Low

Emergency Department at one university hospital, UK	Patient recruitment: 1 January 2013 – 1 March 2013.	Comparator: Current practice at the North-West England University Hospital.		Specificity: 38.9% (95% CI 31.5 to 47.1)<br><br>Positive predictive value: 3% (calculated by CRD)<br><br>Negative predictive value: 100% (95% CI 92.7 to 100)<br><br>Overall accuracy: 40.1% (calculated by CRD)<br><br>**Current practice:**<br><br>Sensitivity: 100% (95% CI 31.0 to 100)<br><br>Specificity: 58.5% (95% CI 50.5 to 66.2)<br><br>Positive predictive value: 4.3% (calculated by CRD)<br><br>Negative predictive value: 100% (95% CI 95.1 to 100)<br><br>Overall accuracy: 59.3% (calculated by CRD)<br><br>Prevalence: 1.9%	Flow/timing: Low<br><br>(limited reporting, as only a conference poster was available)

				**Diagnostic tests performed**<br><br>Based on current practice 42.6% patients were investigated with CT and no patients with SAH were missed. Retrospective application of the Canadian SAH rules to this cohort would have increased the CT investigation rate to 54.3%, 64.8%, 50% and 61.7% for Rule 1, Rule 2, Rule 3 and the Ottawa SAH Rule, respectively (p<0.001). One patient that suffered a SAH would have been missed if Rule 3 was applied.<br><br>**Other significant diagnoses**<br><br>3 patients (1.9%) were diagnosed with SAH by CT, 11 (6.8%) were diagnosed with other cerebral pathologies and 148 (91.4%) were diagnosed with benign causes of headaches.	
Perry, 2017[32]<br><br>Prospective cohort study	1153 non-traumatic, alert, neurologically intact (GCS 15) headache patients (peaking within 1 hour).	Ottawa SAH Rule.	CT, LP (xanthochromia on visual inspection or >1x10^6/L RBCs in the final tube of CSF with aneurysm or arteriovenous malformation seen on angiography) and clinical follow-up (telephone	**Diagnostic accuracy results**<br><br>**Ottawa SAH Rule (SAH):**<br><br>Sensitivity: 100% (95% CI 94.6 to 100)<br><br>Specificity: 13.6% (95% CI 13.1 to 15.8)	Patient selection: Low<br><br>Index test: Low<br><br>Reference standard: Low<br><br>Flow/timing: Low

Emergency Departments at six tertiary care university hospitals, Canada	Patient recruitment: January 2010 – January 2014 (may be patient overlap with Perry, 2010[25]).		follow-up at 1 month and 6 months and medical record review).	Positive predictive value: 6.7% (calculated by CRD)<br><br>Negative predictive value: 100% (calculated by CRD)<br><br>Overall accuracy: 18.6% (calculated by CRD)<br><br>Prevalence: 5.8%<br><br>**Diagnostic tests performed**<br><br>89.1% patients had a CT scan and 39.2% had LP; 37.8% had CT scan and LP. 18% patients had a CT angiogram. 8.6% were admitted to hospital.<br><br>**Other significant diagnoses**<br><br>Final diagnosis: 67 (5.8%) SAH, 8 (0.7%) intracerebral haemorrhage, 6 (0.5%) ischemic stroke or TIA, 3 (0.3%) brain tumour, 3 (0.3%) bacterial meningitis, 2 (0.2%) subdural hematoma. The most common diagnoses were benign headache (53.7%), migraine (19.3), other benign cause (10.4%).	

Bellolio, 2015[28]<br><br>Retrospective cohort study<br><br>Emergency Department at one academic hospital, USA	454 non-traumatic, neurologically intact (GCS 15) headache patients (peaking within 1 hour).<br><br>Patient recruitment: January 2011 – November 2013.	Ottawa SAH Rule.	CT, LP (xanthochromia or RBCs in the final tube of CSF with aneurysm or arteriovenous malformation seen on angiography) and clinical follow-up (medical record review).	**Diagnostic accuracy results**<br><br>**Ottawa SAH Rule (SAH):**<br><br>Sensitivity: 100% (95% CI 62.9 to 100)<br><br>Specificity: 7.6% (95% CI 5.4 to 10.6)<br><br>Positive predictive value: 2.1% (95% CI 1.0 to 4.2)<br><br>Negative predictive value: 100% (95% CI 87.4 to 100)<br><br>Overall accuracy: 9.5% (calculated by CRD)<br><br>Prevalence: 2.0%<br><br>**Diagnostic tests performed**<br><br>79% patients had a CT scan, 17% had LP; 21.9% had LP after negative CT. 10% patients had CT angiogram. Application of the Ottawa SAH Rule at the time of investigation in this cohort would have prevented 13 CTs but would have indicated additional workup in 71 patients with no further yield of SAH cases.	Patient selection: Unclear<br><br>Index test: Unclear<br><br>Reference standard: Unclear<br><br>Flow/timing: Low

				**Other significant diagnoses**<br><br>Final diagnosis: 9 SAH, 7 ischemic stroke or TIA, 1 intracerebral haemorrhage, 1 brain tumour, 1 bacterial meningitis, 1 subdural hematoma.	
Wu, 2019[34]<br><br>Retrospective cohort study<br><br>Emergency Department at one tertiary academic medical centre, Taiwan	913 non-traumatic, neurologically intact patients with a principal diagnosis of headache (time to peak intensity not stated; 8.2% had thunderclap headache).<br><br>Patient recruitment: January 2016 – March 2017.	Ottawa SAH Rule.	Final diagnosis. The authors defined headache secondary to SAH or ICP based on a new neuroimaging finding, such as brain MRI, CT, CSF study, or diagnosed by a neurologist at hospital discharge.	**Diagnostic accuracy results**<br><br>**Ottawa SAH Rule (SAH):**<br><br>Sensitivity: 100% (95% CI 78.2 to 100)<br><br>Specificity: 37% (95% CI 33.8 to 40.2)<br><br>Positive predictive value: 2.6% (95% CI 1.5 to 4.2)<br><br>Negative predictive value: 100% (95% CI 98.9 to 100)<br><br>Overall accuracy: 38% (calculated by CRD)<br><br>Prevalence: 1.6%<br><br>**Ottawa SAH Rule (SAH or intracranial haemorrhage):**	Patient selection: Low<br><br>Index test: Unclear<br><br>Reference standard: High<br><br>Flow/timing: High

				Sensitivity: 100% (95% CI 84.6 to 100)<br><br>Specificity: 37.3% (95% CI 34.1 to 40.5)<br><br>Positive predictive value: 3.8% (95% CI 2.4 to 5.7)<br><br>Negative predictive value: 100% (95% CI 98.9 to 100)<br><br>Overall accuracy: 38.8% (calculated by CRD)<br><br>Prevalence: 2.4%<br><br>**Diagnostic tests performed**<br><br>33.1% patients had a CT scan taken during their ED visit, with an average time to CT ordered of 42.4 ± 73.6 minutes. Patients who received a CT scan had a longer ED length of stay ($p<0.001$)<br><br>**Other significant diagnoses**<br><br>Final diagnosis: 15 (1.6%) SAH, 46 (5.0%) intracranial pathology	

				(including 24 non-haemorhagic intracranial pathology).	
Chu, 2018[30]<br><br>Retrospective cohort study (sub-study of Chu *et al*., 2017, a prospective snapshot of 34 EDs, which was excluded as it also included non-neurologically intact patients)<br><br>34 Emergency Departments in Queensland, Australia	137 non-traumatic headache patients (peaking within 1 hour) with no neurological deficit. The study included 847 patients in total, 137 of which met the Ottawa SAH Rule criteria (and our inclusion criteria).<br><br>Patient recruitment: September 2014.	Ottawa SAH Rule.	Discharge diagnosis (CT or review of state-wide electronic records ≥3 months after presentation).	**Diagnostic accuracy results**<br><br>**Ottawa SAH Rule (SAH):**<br><br>Sensitivity: 100% (calculated by CRD)<br><br>Specificity: 22.4% (calculated by CRD)<br><br>Positive predictive value: 2.8% (calculated by CRD)<br><br>Negative predictive value: 100% (calculated by CRD)<br><br>Overall accuracy: 24.1% (calculated by CRD)<br><br>Prevalence: 2.2% (calculated by CRD)<br><br>**Diagnostic tests performed**<br><br>107 (78.1%) patients had at least one high risk feature on the Ottawa SAH Rule (met work-up criteria); of which 49 had CT head with 3 CTs positive for SAH. Of the 58 patients who met the work-up criteria but did not have CT, none had SAH within 3 months.	Patient selection: Unclear<br><br>Index test: Unclear<br><br>Reference standard: Low<br><br>Flow/timing: Low

				30 (21.9%) patients did not meet work-up criteria, of which 5 had CT head and 25 did not have CT; none of which had SAH within 3 months. 54 (39.4%) patients underwent CT.	
Pathan, 2018 [31]<br><br>Retrospective cohort study<br><br>Emergency Department at one university hospital, UK	145 non-traumatic, alert headache patients (peaking within 1 hour) with no new neurological deficits. The study included 737 patients in total, 145 of which met the Ottawa SAH Rule criteria (and our inclusion criteria) and were included in the analysis of the Ottawa SAH Rule.<br><br>Patient recruitment: 1 January 2016 – 31 December 2016.	Ottawa SAH Rule.<br><br>Comparator: Current practice without a rule assessed in all headache patients (including those not meeting our inclusion criteria).	CT and/or LP (subarachnoid blood on CT or xanthochromia in the CSF).	**Diagnostic accuracy results**<br><br>**Ottawa SAH Rule (SAH):**<br><br>Sensitivity: 100% (95% CI 46.3 to 100)<br><br>Specificity: 44.2% (95% CI 36 to 53)<br><br>Positive predictive value: 6% (95% CI 2.2 to 14.1)<br><br>Negative predictive value: 100% (95% CI 92.7 to 100)<br><br>Overall accuracy: 46.2% (calculated by CRD)<br><br>Prevalence: 3.4%<br><br>Diagnostic accuracy results were also presented for current practice without a rule, but not all patients met our inclusion criteria.	Patient selection: Low<br><br>Index test: Unclear<br><br>Reference standard: Low<br><br>Flow/timing: Unclear

				**Diagnostic tests performed**<br><br>87 (60%) patients who met Ottawa SAH Rule criteria had a CT scan. 35 (24%) patients who met Ottawa SAH Rule criteria had a LP. According to the Ottawa SAH Rule 62 patients required no further investigations and 83 required further work-up with CT ± LP.	
Cheung, 2018[29]<br><br>Retrospective cohort study<br><br>Emergency Department at one regional hospital, Hong Kong	500 non-traumatic, neurologically intact (GCS 15), acute headache patients (peaking within 1 hour).<br><br>Patient recruitment: July 2013 – June 2016.	Ottawa SAH Rule (validation in Asian Chinese patients).<br><br>Comparator: Modified Ottawa SAH Rule including both vomiting and SBP >160 mmHg.	CT (films reviewed by both an experienced emergency physician and radiology fellow), LP (xanthochromia or RBCs in the final tube of CSF with aneurysm or arteriovenous malformation seen on angiography).	**Diagnostic accuracy results**<br><br>**Ottawa SAH Rule (SAH):**<br><br>Sensitivity: 94% (95% CI 82.5 to 98.4)<br><br>Specificity: 32.9% (95% CI 28.6 to 37.5)<br><br>Positive predictive value: 13.5% (95% CI 10.2 to 17.6)<br><br>Negative predictive value: 98% (95% CI 93.9 to 99.5)<br><br>Overall accuracy: 39% (calculated by CRD)<br><br>**Modified Ottawa SAH Rule (SAH):**	Patient selection: Low<br><br>Index test: Unclear/High<br><br>Reference standard: Low<br><br>Flow/timing: Low

				Sensitivity: 100% (95% CI 91.1 to 100)<br><br>Specificity: 13.1% (95% CI 10.2 to 16.7)<br><br>Positive predictive value: 11.3% (95% CI 8.6 to 14.8)<br><br>Negative predictive value: 100% (95% CI 92.4 to 100)<br><br>Overall accuracy: 21.8% (calculated by CRD)<br><br>Prevalence: 10% (34/50 SAH patients had aneurysmal SAH)<br><br>**Diagnostic tests performed**<br><br>96.2% patients had a CT scan and 10% had LP.	
Perry, 2020[33]<br><br>Prospective before/after implementation study	3672 non-traumatic, alert patients (GCS 15) with acute headache or headache-associated syncope (peaking within 1 hour).	Physician education to use Ottawa SAH Rule and 6-hour-CT rule.<br><br>Comparator: Control period (before implementation).	CT (3rd generation or better using thin slices), LP (xanthochromia on visual inspection or >1x10^6/L RBCs in the final tube of CSF with aneurysm seen on angiography) and clinical follow-up (electronic	**Diagnostic accuracy results**<br><br>**Ottawa SAH Rule (SAH):**<br><br>Sensitivity: 100% (95% CI 98.1 to 100)<br><br>Specificity: 12.7% (95% CI 11.7 to 13.9)	Patient selection: Low<br><br>Index test: Low<br><br>Reference standard: Low<br><br>Flow/timing: Low

Emergency Departments at six academic hospitals, Canada<br><br>*Also reported in CT scan section*	Patient recruitment: January 2010 – June 2013 (before implementation) and June 2013 – January 2016 (after implementation).		health record review at 6 months and study end).	Positive predictive value: 5.8% (calculated by CRD)<br><br>Negative predictive value: 100% (calculated by CRD)<br><br>Overall accuracy: 17.2% (calculated by CRD)<br><br>Prevalence: 5.1%<br><br>**6-hour-CT Rule (SAH):**<br><br>1204 patients received CT within 6 hours<br><br>Sensitivity: 95.5% (95% CI 89.8 to 98.5)*<br><br>Specificity: 100% (95% CI 99.7 to 100)<br><br>Positive predictive value: 100% (calculated by CRD)<br><br>Negative predictive value: 99.5% (calculated by CRD)<br><br>Overall accuracy: 99.6% (calculated by CRD)<br><br>Prevalence: 9.2% (calculated by CRD)	

				*5 patients had SAH with CT reported as normal: 2 unruptured aneurysms on CTA and presumed traumatic LP; 1 missed by the radiologist on initial interpretation; 1 dural vein fistula (i.e. nonaneurysmal); and 1 patient with sickle cell anaemia with profound anaemia (Hgb, 63 g/L) with a 3mm aneurysm.<br><br>6-hour-CT Rule (SAH) with 2 incidental aneurysms reclassified as true negatives:<br><br>Sensitivity: 97.2% (95% CI 94.2 – 100) (calculated by CRD)<br><br>**Diagnostic tests performed**<br><br>The rate of CT use remained constant; 88.0% in the control phase vs 87.5% in the intervention phase. The LP rate decreased from 38.9% to 25.9% (p<0.0001). The CTA rate increased from 18.8% to 21.7% (p=0.029). Admission rates decreased from 9.8% to 7.4% (p=0.011). Time from Emergency Physician assessment to discharge/referral was	

				slightly longer (4.9 hours vs 5.2 hours; p=0.053). Mean length of stay in the ED was similar 6.3 vs 6.4 hours; p=0.685).<br><br>**Other significant diagnoses**<br><br>Final diagnosis: 188 (5.1%) SAH, 26 (0.7%) ischemic stroke or TIA, 24 (0.7%) intracerebral haemorrhage, 10 (0.3%) brain tumour, 7 (0.2%) bacterial meningitis.	
**Pathway of CT followed by LP**					
Perry, 2002[38]<br><br>Retrospective cohort study<br><br>Emergency Department at one tertiary care university centre, Canada	891 non-traumatic, alert patients (GCS 15) with acute headache or syncope (peaking within 1 hour).<br><br>Patient recruitment: 1 January 2000 – 31 October 2000.	Pathway of CT followed by LP.	Not applicable.	**Length of stay**<br><br>Mean ED length of stay was 239 minutes (SD 148.3, range 17-1438 minutes). The mean ED length of stay was 4 hours (95% CI 3.8 to 4.1) if no diagnostic testing was performed, 5 hours (95% CI 4.7 to 5.4) if CT was performed and 7.1 hours (95% CI 6.3 to 7.9) if LP was performed.<br><br>**Diagnostic tests performed**<br><br>313 (35.1%) patients underwent CT; 9 were positive for SAH and 8 were positive for other acute processes	Unclear

				(neoplasm or infarct). 85/891 (9.5%) patients underwent LP; 2 were positive for SAH (one of which had a positive CT result before LP, the other had LP without CT). 64/296 (21.6%) underwent LP after negative CT.<br><br>**Other significant diagnoses**<br><br>32 (3.6%) patients had potentially dangerous conditions: 10 (1.1%) SAH, 9 ischemic event, 6 brain tumour, 4 bacterial meningitis, 3 temporal arteritis. The most common diagnoses were migraine (43.7%), other benign headache (33.1%) and other/not determined (10.7%). 426 (2.9%) patients were referred to the neurosurgical service and 33 (3.7%) were admitted.	
Perry, 2008[39]<br><br>Prospective cohort study<br><br>Emergency departments at two	592 non-traumatic, alert, neurologically intact (GCS 15) headache patients (peaking within 1 hour) or syncope associated with headache.	CT (using final neuroradiology report), followed by LP if CT negative (visual inspection of CSF for xanthochromia or >5x10^6 RBCs/L in the final tube).	SAH defined by CT (using final neuroradiology report), LP (xanthochromia on visual inspection or >5x10^6/L RBCs in the final tube of CSF with aneurysm seen on angiography) or autopsy report confirming SAH.	**Diagnostic accuracy results**<br><br>**CT followed by LP (SAH):**<br><br>Sensitivity: 100% (95% CI 94 to 100)<br><br>Specificity: 67% (95% CI 63 to 71)<br><br>Positive predictive value: 25.8% (calculated by CRD)	Patient selection: Low<br><br>Index test: Low<br><br>Reference standard: Low<br><br>Flow/timing: Low

tertiary care hospitals, Canada	Patient recruitment: November 2000 – November 2003 (appears to be patient overlap with Perry, 2011[43]).		Patients were contacted via telephone to verify that they had not had subsequent adverse events or diagnosis of SAH.	Negative predictive value: 100% (95% CI 98 to 100)<br><br>Overall accuracy: 70.4% (calculated by CRD)<br><br>Prevalence: 10.3%<br><br>55/61 SAH cases were diagnosed on CT, 6 by presence of xanthochromia.<br><br>**Diagnostic tests performed**<br><br>100% patients underwent CT, 91% underwent LP and 13% underwent angiography. 68 patients (11.5%) had an abnormal CT result and 183 (34.0%) had an abnormal LP result; xanthochromia was detected in the CSF of 7 patients (1.2%).<br><br>**Other significant diagnoses**<br><br>Other significant pathologies detected were transient ischemic attack (0.8%), bacterial meningitis (0.2%), CNS tumour (0.2%) and intracerebral haemorrhage (0.2%). The most common diagnoses were	

				benign headache (46.5%) and migraine (26.4%).	
Valle Alonso, 2018[40]<br><br>Retrospective cohort study<br><br>Emergency Department at one regional hospital, Spain<br><br>*Also reported in CT scan section and Lumbar puncture section*	85 non-traumatic, sudden headache patients (peaking within 1 hour) without unconsciousness or neurological focus, presenting to the ED within 6 hours of symptom onset.<br><br>Patient recruitment: March 2012 – March 2013.	CT (within 6 hours) followed by LP, if CT negative for SAH.<br><br>The CT used was multi-slice (4-320 slices/rotation) with slices of 5 - 7.5 mm for the brain and 2.5 – 5 mm for the posterior fossa. The CT report was made by deputies of the radiology service, with over 5 years of experience and in consultation with the neuroradiologist when there was doubt.	LP was performed in all patients with a negative CT scan. Clinical follow-up at 6 months using medical records or phone calls where there was no conclusive data in medical records.	**Diagnostic accuracy results**<br><br>**CT within 6 hours (SAH):**<br><br>Sensitivity: 100% (calculated by CRD)<br><br>Specificity: 98.7% (calculated by CRD)<br><br>Positive predictive value: 90.9% (calculated by CRD)<br><br>Negative predictive value: 100% (calculated by CRD)<br><br>Overall accuracy: 98.8% (calculated by CRD)<br><br>Prevalence: 11.8% (calculated by CRD)<br><br>**Diagnostic tests performed**<br><br>74 (87%) patients underwent LP; LP was positive in 1 patient and inconclusive in 2 patients. However, bleeding was ruled out with later images; thus no cases of SAH were identified by LP. No cases of SAH were reported during the 6 months of	Patient selection: Unclear<br><br>Index test: Low<br><br>Reference standard: Low<br><br>Flow/timing: Low

				follow-up. 7 patients experienced post puncture headache, going back to the ED and admission was necessary for 2 of them for pain control.<br><br>**Other significant diagnoses**<br><br>The most frequent final diagnosis was migraine (38.8%). 9.4% had a severe diagnosis, such as meningitis (4.7%) and reversible cerebral vasoconstriction syndrome (4.7%).<br><br>**SAH patient signs and symptoms**<br><br>SAH patients were more likely to arrive at ED by ambulance (p=0.010) and have occipital headache location (p=0.012). Among the clinical signs highlighted, the presence of syncope (p=0.036), neck pain or stiffness (p=0.010), photophobia (p=0.001), nausea or vomiting (p=0.000), as well as higher numbers of systolic (mean 153 vs 126) and diastolic blood pressure (mean of 100 vs 80) (p=0.000).	

Cooper, 2016[36]<br><br>Retrospective cohort study<br><br>Clinical Decision Unit at one teaching hospital, UK<br><br>*Also reported in CT scan section and Lumbar puncture section*	517 non-traumatic, neurologically pristine (GCS 15) patients with acute sudden onset severe headache managed on a CDU pathway for exclusion of SAH.<br><br>Patient recruitment: January 2004 – December 2006.	CDU pathway of CT followed by LP.<br><br>Initial and verified non-contrast CT reports (performed on third-generation scanners) and LP results (all taken >12 hours from the index headache).	CT (verified by a consultant radiologist), LP (CSF positive for bilirubin on spectrophotometry or a uniformly blood-stained CSF sample across four bottles and positive angiography). If CT/LP strategy was not completed, sudden death or subsequent SAH was assessed at 12 months by analysing attendance and investigations (electronic hospital database).	**Diagnostic accuracy results**<br><br>**CT (SAH):**<br><br>Sensitivity: 92.9% (95% CI 79.5 to 100)<br><br>Specificity: 100% (95% CI 99.6 to 100)<br><br>Positive predictive value: 100% (95% CI 98.2 to 100)<br><br>Negative predictive value: 99.8% (95% CI 99.4 to 100)<br><br>Overall accuracy: 99.8% (calculated by CRD)<br><br>Prevalence: 2.7% (14/510 who had CT)<br><br>**LP after negative CT (SAH):**<br><br>Sensitivity: 100% (95% CI 93.7 to 100)<br><br>Specificity: 96.8% (95% CI 94.8 to 98.8)<br><br>Positive predictive value: 9.1% (95% CI 0 to 26.1)	Patient selection: Low<br><br>Index test: Unclear<br><br>Reference standard: Low<br><br>Flow/timing: Unclear

				Negative predictive value: 100% (95% CI 99.5 to 100)<br><br>Overall accuracy: 96.8% (calculated by CRD)<br><br>Prevalence: 0.3% (1/309 who had LP)<br><br>CT was positive for SAH in 13 patients; 6 had an underlying lesion on angiography and 7 had perimesencephalic SAH. 4 CT scans were initially reported as 'normal' making patients eligible for LP, only to be subsequently altered in 3 cases to SAH positive after neuroradiological interpretation of the CT scan.<br><br>LP was positive for SAH in 11 patients; 10 patients were LP positive but angiography negative (false positives).<br><br>**Diagnostic tests performed**<br><br>510 (98.6%) patients had a CT scan and 309 had LP. 491 patients were eligible for LP (490 initially negative	

				on CT + 1 patient who went straight to LP without CT); 182 eligible patients did not have LP due to procedure failure (n=18), patient refusal or contraindication (n=65) or decision of attending doctor (n=99).<br><br>**Other significant diagnoses**<br><br>CT was positive for other significant aetiology in a further 14 patients: 4 cerebral infarction, 2 venous sinus thrombosis, 2 incidental cerebral aneurysm, 1 arachnoid cyst, 1 metastatic disease, 1 haemangioma, 1 subdural haemorrhage, 1 meningioma, 1 bleed into glioblastoma. LP was positive for other significant aetiology in a further 17 patients: 16 viral meningitis and 1 nonocclusive sagital sinus thrombosis.	
Blok, 2015[35]<br><br>Retrospective cohort study	760 neurologically intact (GCS 15) 'spontaneous' acute headache patients with suspected SAH, who underwent CT within 6 hours of onset (judged negative by radiologist) and subsequent LP.	CT (third generation scanner) <6 hours from headache onset (assessed by a staff radiologist), followed by LP >12 hours after onset (CSF was analysed using spectrophotometry).	Review of admission CTs in patients with bilirubin positive CSF by two neuroradiologists and one stroke neurologist. Lumbar puncture >12 hours after onset (CSF was analysed using spectrophotometry using	**Diagnostic accuracy results**<br><br>52 (7%) CSF samples were initially considered positive for SAH, but only one CT was positive for subarachnoid blood (in the basal cisterns) on review by two neuroradiologists and one stroke neurologist; angiography did not identify an aneurysm and the patient	Patient selection: Low<br><br>Index test: Low<br><br>Reference standard: Unclear<br><br>Flow/timing: Unclear

Emergency Departments at eleven non-academic hospitals, Netherlands<br><br>*Also reported in CT scan section*	Patient recruitment: January 2007 – January 2013.		a number of methods across the 11 sites: oxyhaemoglobin/bilirubin concentration, UK NEQAS, qualitative assessment of absorption curve, Leiden method, and bilirubin excess).	was diagnosed with non-aneurysmal perimesencephalic haemorrhage (with a benign clinical course and no readmission for SAH during 26 month follow-up). No subarachnoid blood was identified in the other 51 patients with positive CSF findings. 28/51 patients had angiography; aneurysm was identified in 8 patients (3 previously coiled). In those with an aneurysm it was considered that aneurysm rupture was unlikely and the aneurysm was considered incidental (4 were treated and 4 were not).<br><br>The negative predictive value for detection of subarachnoid blood on CT by staff radiologists working in a non-academic hospital was 99.9% (95% CI 99.3 to 100). SAH prevalence was 0.13% (1/760).	
Dutto, 2009[37]<br><br>Before and after study	70 non-traumatic, neurologically intact (GCS 15), alert patients with headache (25 before and 45 after implementation of the intervention). The study included 686 patients in total, patients were	Diagnostic protocol for non-traumatic acute headache in the ED, there was a different flow chart for each of the 3 subgroups. The flow chart recommended LP (if deemed necessary) for patients who had a negative CT scan result but who were suspected of	Not applicable.	**Diagnostic tests performed**<br><br>43/45 (95.5%) patients underwent CT scan after implementation of the diagnostic protocol versus 24/25 (96%) before. 2 patients received LP; both were negative. Neurological consultations were performed in 30/45 (66.6%) patients	Unclear

Emergency Department at one urban non-teaching hospital, Italy	retrospectively assigned to 3 subgroups based on headache characteristics; subgroup 1 comprised patients with suspected SAH; thunderclap headache, 'worst headache ever', neurological signs, syncope, vomiting/nausea or onset following exertion (who met our inclusion criteria).<br><br>Patient recruitment: April – September 2005 (before) and April – September 2006 (after).	SAH. Where SAH was not suspected or where LP results were normal, the attending physician could consult a neurologist for further clinical decisions.<br><br>Comparator: Normal practice pre-implementation.		after the intervention versus 19/25 (76.0%) before.<br><br>In the full population, the protocol was strictly applied in 247/374 (66%) patients after implementation. A higher proportion of patients received neither a CT scan nor a neurological consultation after implementation of the protocol than before (40.9% versus 34%). Patients spent less time in the ED after implementation of the protocol than before (170.6 ± 102 minutes versus 180 ± 105 minutes).<br><br>**Significant diagnoses**<br><br>Malignant secondary headaches (including SAH, neoplasm, intracranial haemorrhage and ischemic stroke) were diagnosed in 30/686 (4.37%) patients in the full population, with SAH accounting for 10 cases (1.5%); 5 before and 5 after implementation of the protocol. There was 1 misdiagnosis (cerebral neoplasm) after the intervention and two misdiagnoses (1 SAH, 1 intracerebral haemorrhage) before the intervention.	

**CT scan**					
Perry, 2010[25]<br><br>Prospective cohort study<br><br>Emergency Departments at six university affiliated tertiary care teaching hospitals, Canada<br><br>*Also reported in Canadian clinical decision rules section*	1999 non-traumatic, alert, neurologically intact (GCS 15) headache patients (peaking within 1 hour) or syncope associated with headache. An additional 1050 potentially eligible patients were identified who were not enrolled 'missed eligible patients'.<br><br>Patient recruitment: November 2000 – November 2005 (patient overlap with Perry, 2011[43])	Third generation CT scanner, results verified by the local attending radiologist (either neuroradiologists or general radiologists who routinely interpret head CT).<br><br>Identification of high risk clinical characteristics for SAH in order to develop clinical decision rules based on variables collected on history or examination.<br><br>Rule 1: age >40; complaint of neck pain or stiffness; witnessed loss of consciousness; onset with exertion.<br><br>Rule 2: arrival by ambulance; age >45; vomiting at least once; diastolic BP >100 mm Hg.	CT, LP (xanthochromia on visual inspection or >5x10^6/L RBCs in the final tube of CSF with aneurysm or arteriovenous malformation seen on angiography) and clinical follow-up (telephone follow-up at 1 month and 6 months and medical record review).	**Diagnostic accuracy results**<br><br>**CT (SAH):**<br><br>Sensitivity: 93.1% (calculated by CRD)<br><br>Specificity: 100% (calculated by CRD)<br><br>Positive predictive value: 100% (calculated by CRD)<br><br>Negative predictive value: 99.4% (calculated by CRD)<br><br>Overall accuracy: 99.4% (calculated by CRD)<br><br>Prevalence: 6.5%<br><br>**Clinical decision rules (SAH):**<br><br>Retrospective sensitivity: Rule 1-3: 100% (95% CI 97.1 to 100)<br><br>Specificity: Rule 1: 28.4% (95% CI 26.4 to 30.4); Rule 2: 36.5% (95% CI 34.4 to 38.8); Rule 3: 38.8% (95% CI 36.7 to 41.1).	Patient selection: Unclear<br><br>Index test: Low<br><br>Reference standard: Low<br><br>Flow/timing: Low

		Rule 3: arrival by ambulance; systolic BP >160 mm Hg; complaint of neck pain or stiffness; age 45-55.		**Diagnostic tests performed**<br><br>1606 (80.3%) patients had a CT scan and 905 (45.3%) had LP; 854 (42.7%) had CT scan and LP. 8.4% patients had a CT angiogram. Use of any one of the rules assessed would have lowered rates of investigation (CT, LP or both) from 82.9% to between 63.7-73.5%.<br><br>**Other significant diagnoses**<br><br>48 patients had other serious conditions diagnosed on CT or LP, such as transient ischaemic attack/acute ischaemic stroke, other type of haemorrhagic stroke, bacterial meningitis, hypertensive emergency or cerebral neoplasm.	
Perry, 2011[43]<br><br>Prospective cohort study (part of a larger project on clinical decision rules: Perry, 2010[25])	3132 non-traumatic, alert, neurologically intact (GCS 15) headache patients (peaking within 1 hour) or syncope associated with headache, who underwent CT as part of their diagnostic intervention.	Third generation multi-slice CT scanner (from 4 to 320 slices/rotation), interpreted by local radiologists (either neuroradiologists or general radiologists who routinely interpret head CT). The final local 'sign off' report was used, even though it might be created the next day, especially when the scan was obtained during the evening or	CT, LP (xanthochromia on visual inspection or >5x10^6/L RBCs in the final tube of CSF with aneurysm or arteriovenous malformation seen on angiography) and clinical follow-up (telephone follow-up at 1 month and	**Diagnostic accuracy results**<br><br>**CT overall (SAH):**<br><br>Sensitivity: 92.9% (95% CI 89.0 to 95.5)<br><br>Specificity: 100% (95% CI 99.9 to 100)<br><br>Positive predictive value: 100% (95% CI 98.3 to 100)	Patient selection: Low<br><br>Index test: Low<br><br>Reference standard: Low<br><br>Flow/timing: Low

Emergency Departments at eleven university affiliated tertiary care teaching hospitals, Canada	Patient recruitment: November 2000 – December 2009 (patient overlap with Perry, 2010[25]).	weekend. The protocols at the beginning of the study (2000-2002) used 5 mm slices for the posterior fossa and 10 mm for the remainder of the brain. Since 2002 all sites adopted 5-7.5 mm cuts for the brain with 2.5-5 mm for the posterior fossa.	6 months and medical record review).	Negative predictive value: 99.4% (calculated by CRD)<br><br>Overall accuracy: 99.5% (calculated by CRD)<br><br>Prevalence: 7.7%<br><br>**CT within 6 hours of symptom onset (SAH):**<br><br>Sensitivity: 100% (95% CI 97.0 to 100)<br><br>Specificity: 100% (95% CI 99.5 to 100)<br><br>Positive predictive value: 100% (95% CI 96.9 to 100)<br><br>Negative predictive value: 100% (95% CI 99.5 to 100)<br><br>Overall accuracy: 100% (calculated by CRD)<br><br>Prevalence: 12.7%<br><br>**CT >6 hours from symptom onset (SAH):**	

				Sensitivity: 85.7% (95% CI 78.3 to 90.9)<br><br>Specificity: 100% (95% CI 99.8 to 100)<br><br>Positive predictive value: 100% (calculated by CRD)<br><br>Negative predictive value: 99.2% (calculated by CRD)<br><br>Overall accuracy: 99.2% (calculated by CRD)<br><br>Prevalence: 4.7%<br><br>**Diagnostic tests performed**<br><br>3132 (100%) patients had a CT scan; 953 (30.4%) within 6 hours of symptom onset. 1546/3132 (49.4%) had LP.<br><br>3 SAH patients were discharged after misinterpretation of the CT scan by emergency physicians, but were recalled after review of the CT by radiologists. One CT was initially misinterpreted as normal by the emergency physician and radiology trainee; the patient had blood in the	

				CSF attributed to traumatic LP and was found to have an aneurysm on follow-up MR angiogram 5 days later.	
Khan, 2017[42]<br><br>A priori planned secondary analysis of two sequential prospective cohort studies<br><br>Emergency Departments at eleven university affiliated hospitals, Canada	2412 non-traumatic, neurologically intact (GCS 15) acute headache patients (peaking within 1 hour). 3315 patients were recruited in total, but only 2412 had complete information.<br><br>Same cohort of patients as Perry, 2010[25] and Perry, 2011.[43]<br><br>Patient recruitment: 2000 – 2010.	CT, results determined by an experienced radiologist (either a neuroradiologist or general radiologist who regularly interprets head CT images).	CT, LP (xanthochromia on visual inspection or >5x10^6/L RBCs in the final tube of CSF with aneurysm seen on angiography) and clinical follow-up (telephone follow-up at 14 days and medical record review).	**Diagnostic accuracy results**<br><br>194 (8.0%) patients had a final diagnosis of SAH; 178/194 cases (91.8%) were identified using CT (91.8% sensitivity).<br><br>727 patients had CT within 6 hours of headache onset; 91 (12.5%) had SAH; all cases were identified using CT (100% sensitivity).<br><br>1685 patients had CT over 6 hours from headache onset; 103 (6.1%) had SAH; 87/103 (84.5%) were identified using CT (84.5% sensitivity).<br><br>**Diagnostic tests performed**<br><br>100% patients had a CT scan, 1222 (50.7%) patients had LP and 206 (8.5%) had angiography. 273 (11.3%) patients were admitted to hospital; 180 SAH patients and 93	Patient selection: Low<br><br>Index test: Low<br><br>Reference standard: Low<br><br>Flow/timing: Low

				non-SAH patients. 11 (0.5%) patients died; all had SAH.<br><br>Median time from headache onset to CT was significantly shorter for patients with SAH; 6.4 hours (IQR 3.5 – 27.1) versus 12.6 hours (IQR 5.5 – 48.0) for those without SAH (p<0.001). Most of this difference was due to SAH patients presenting to hospital earlier on average than non-SAH patients (4.5 hours (IQR 1.7-22.7) vs 9.6 hours (IQR 2.8-46.0), p<0.001). The in-hospital interval from registration to imaging was also significantly shorter in SAH patients (1.9 hours (IQR 1.2-2.8) vs 2.5 hours (IQR 1.5-3.9), p<0.001).<br><br>**SAH patient signs and symptoms**<br><br>Patients with SAH were older (52.7 vs 44.2 years, p<0.001), were more likely to have arrived by ambulance (56.2% vs 21.7%, p<0.001), vomited (65.5% vs 26.8%, p<0.001) and experienced witnessed loss of consciousness (7.7% vs 3.2%, p<0.001).	

Backes, 2012[41]<br><br>Retrospective cohort study<br><br>Emergency department at one university hospital, Netherlands	250 non-traumatic, alert, neurologically intact (GCS 15) headache patients with a clinical suspicion of SAH. Patients were identified from databases of SAH patients and patients in whom SAH was ruled out using CT and LP. 247/250 (98.8%) experienced headache.<br><br>Patient recruitment: 1 January 2005 – 1 January 2012 (likely patient overlap with Backes, 2015[58]).	Plain head CT scan (16-256 slices per rotation multidetector row third-generation scanner with a slice thickness of 5 mm). CT scans were interpreted by an experienced neuroradiologist.<br><br>Head CT was performed within 6 hours of symptom onset in 137 patients (54.8%) and >6 hours in 113 patients (45.2%).	LP performed ≥12 hours after ictus (CSF was examined using visual inspection and spectrophotometry for the presence of bilirubin).	**Diagnostic accuracy results**<br><br>**CT overall (aSAH):**<br><br>Sensitivity: 95.4% (95% CI 89.5 to 98.5)<br><br>Specificity: 100% (95% CI 97.4 to 100)<br><br>Positive predictive value: 100% (95% CI 96.5 to 100)<br><br>Negative predictive value: 96.6% (95% CI 92.2 to 98.9)<br><br>Overall accuracy: 98.4% (calculated by CRD)<br><br>Prevalence: 35.2% (calculated by CRD)<br><br>**CT within 6 hours of symptom onset (aSAH or other significant pathology*):**<br><br>Sensitivity: 98.5% (95% CI 92.1 to 100)<br><br>Specificity: 100% (95% CI 94.8 to 100)<br><br>Positive predictive value: 100% (95% CI 94.6 to 100)	Patient selection: Low<br><br>Index test: Low<br><br>Reference standard: Low<br><br>Flow/timing: Low

				Negative predictive value: 98.6% (95% CI 92.3 to 100)<br><br>Overall accuracy: 99.3% (calculated by CRD)<br><br>Prevalence: 50.5% (calculated by CRD)<br><br>*perimesencephalic haemorrhage, cerebral venous sinus thrombosis or cervical arteriovenous malformation<br><br>**CT >6 hours from symptom onset (aSAH or other significant pathology*):**<br><br>Sensitivity: 90.0% (95% CI 76.3 to 97.2)<br><br>[88.1% (calculated by CRD)]<br><br>Specificity: 100% (95% CI 95.1 to 100)<br><br>Positive predictive value: 100% (95% CI 90.3 to 100)<br><br>Negative predictive value: 94.8% (95% CI 87.2 to 98.6)<br><br>[93.4% (calculated by CRD)]	

				Overall accuracy: 95.6% (calculated by CRD)<br><br>Prevalence: 37.2% (calculated by CRD)<br><br>*perimesencephalic haemorrhage, acute ischemic stroke or thoracic arteriovenous malformation<br><br>Final diagnosis in those who had CT scan within 6 hours of symptom onset (n=137): 56 aSAH, 11 perimesencephalic haemorrhage and 1 cerebral venous sinus thrombosis. 69 patients with negative/inconclusive CT results had LP; no further aSAH diagnoses but 1 patient was diagnosed with cervical arteriovenous malformation.<br><br>Final diagnosis in those who had CT scan >6 hours from symptom onset (n=113): 28 aSAH, 8 perimesencephalic haemorrhage, 1 acute ischaemic stroke. 76 patients with negative/inconclusive CT results had LP; there were 4 further aSAH diagnoses and 1 cervical arteriovenous malformation. The 4 patients with negative or	

				inconclusive CT results and aSAH on LP had been scanned between 27 hours and 10 days of symptom onset.<br><br>In headache patients (n=247/250), sensitivity of head CT in patients scanned within 6 hours of symptom onset was 100% (95% CI 94.6 to 100), specificity was 100% (95% CI 94.8 to 100). Sensitivity of head CT >6 hours after symptom onset was 92.3% (95% CI 79.1 to 98.4), specificity was 100% (95% CI 95.1 to 100).	
Perry, 2020[33]<br><br>Prospective before/after implementation study<br><br>Emergency Departments at six academic hospitals, Canada	3672 non-traumatic, alert patients (GCS 15) with acute headache or headache-associated syncope (peaking within 1 hour).<br><br>Patient recruitment: January 2010 – June 2013 (before implementation) and June 2013 – January 2016 (after implementation).	Physician education to use Ottawa SAH Rule and 6-hour-CT rule.<br><br>Comparator: Control period (before implementation).	CT (3rd generation or better using thin slices), LP (xanthochromia on visual inspection or >1x10^6/L RBCs in the final tube of CSF with aneurysm seen on angiography) and clinical follow-up (electronic health record review at 6 months and study end).	**Diagnostic accuracy results**<br><br>**Ottawa SAH Rule (SAH):**<br><br>Sensitivity: 100% (95% CI 98.1 to 100)<br><br>Specificity: 12.7% (95% CI 11.7 to 13.9)<br><br>Positive predictive value: 5.8% (calculated by CRD)<br><br>Negative predictive value: 100% (calculated by CRD)<br><br>Overall accuracy: 17.2% (calculated by CRD)	Patient selection: Low<br><br>Index test: Low<br><br>Reference standard: Low<br><br>Flow/timing: Low

Also reported in Canadian clinical decision rules section				Prevalence: 5.1% (calculated by CRD)<br><br>**6-hour-CT Rule (SAH):**<br><br>1204 patients received CT within 6 hours<br><br>Sensitivity: 95.5% (95% CI 89.8 to 98.5)<br><br>Specificity: 100% (95% CI 99.7 to 100)<br><br>Positive predictive value: 100% (calculated by CRD)<br><br>Negative predictive value: 99.5% (calculated by CRD)<br><br>Overall accuracy: 99.6% (calculated by CRD)<br><br>Prevalence: 9.2% (calculated by CRD)<br><br>**Diagnostic tests performed**<br><br>The rate of CT use remained constant; 88.0% in the control phase vs 87.5% in the intervention phase. The LP rate decreased from 38.9% to	

				25.9% (p<0.0001). The CTA rate increased from 18.8% to 21.7% (p=0.029). Admission rates decreased from 9.8% to 7.4% (p=0.011). Time from Emergency Physician assessment to discharge/referral was slightly longer (4.9 hours vs 5.2 hours; p=0.053). Mean length of stay in the ED was similar 6.3 vs 6.4 hours; p=0.685).<br><br>**Other significant diagnoses**<br><br>Final diagnosis: 188 (5.1%) SAH, 26 (0.7%) ischemic stroke or TIA, 24 (0.7%) intracerebral haemorrhage, 10 (0.3%) brain tumour, 7 (0.2%) bacterial meningitis.	
Valle Alonso, 2018[40]<br><br>Retrospective cohort study<br><br>Emergency Department at one regional hospital, Spain	85 non-traumatic, sudden headache patients (peaking within 1 hour) without unconsciousness or neurological focus, presenting to the ED within 6 hours of symptom onset.	CT (within 6 hours) followed by LP, if CT negative for SAH.<br><br>The CT used was multi-slice (4-320 slices/rotation) with slices of 5 - 7.5 mm for the brain and 2.5 – 5 mm for the posterior fossa. The CT report was made by deputies of the radiology service, with over 5 years of experience and in	LP was performed in all patients with a negative CT scan. Clinical follow-up at 6 months using medical records or phone calls where there was no conclusive data in medical records.	**Diagnostic accuracy results**<br><br>**CT within 6 hours (SAH):**<br><br>Sensitivity: 100% (calculated by CRD)<br><br>Specificity: 98.7% (calculated by CRD)<br><br>Positive predictive value: 90.9% (calculated by CRD)	Patient selection: Unclear<br><br>Index test: Low<br><br>Reference standard: Low<br><br>Flow/timing: Low

Also reported in Pathway of CT followed by LP section and Lumbar puncture section	Patient recruitment: March 2012 – March 2013.	consultation with the neuroradiologist when there was doubt.		Negative predictive value: 100% (calculated by CRD)<br><br>Overall accuracy: 98.8% (calculated by CRD)<br><br>Prevalence: 11.8% (calculated by CRD)<br><br>**Diagnostic tests performed**<br><br>74 (87%) patients underwent LP; LP was positive in 1 patient and inconclusive in 2 patients. However, bleeding was ruled out with later images; thus no cases of SAH were identified by LP. No cases of SAH were reported during the 6 months of follow-up. 7 patients experienced post puncture headache, going back to the ED and admission was necessary for 2 of them for pain control.<br><br>**Other significant diagnoses**<br><br>The most frequent final diagnosis was migraine (38.8%). 9.4% had a severe diagnosis, such as meningitis (4.7%) and reversible cerebral vasoconstriction syndrome (4.7%).	

				**SAH patient signs and symptoms**<br><br>SAH patients were more likely to arrive at ED by ambulance (p=0.010) and have occipital headache location (p=0.012). Among the clinical signs highlighted, the presence of syncope (p=0.036), neck pain or stiffness (p=0.010), photophobia (p=0.001), nausea or vomiting (p=0.000), as well as higher numbers of systolic (mean 153 vs 126) and diastolic blood pressure (mean of 100 vs 80) (p=0.000).	
Cooper, 2016[36]<br><br>Retrospective cohort study<br><br>Clinical Decision Unit at one teaching hospital, UK<br><br>*Also reported in Pathway of CT followed by LP*	517 non-traumatic, neurologically pristine (GCS 15) patients with acute sudden onset severe headache managed on a CDU pathway for exclusion of SAH.<br><br>Patient recruitment: January 2004 – December 2006.	CDU pathway of CT followed by LP.<br><br>Initial and verified non-contrast CT reports (performed on third-generation scanners) and LP results (all taken >12 hours from the index headache).	CT (verified by a consultant radiologist), LP (CSF positive for bilirubin on spectrophotometry or a uniformly blood-stained CSF sample across four bottles and positive angiography). If CT/LP strategy was not completed, sudden death or subsequent SAH was assessed at 12 months by analysing attendance and investigations (electronic hospital database).	**Diagnostic accuracy results**<br><br>**CT (SAH):**<br><br>Sensitivity: 92.9% (95% CI 79.5 to 100)<br><br>Specificity: 100% (95% CI 99.6 to 100)<br><br>Positive predictive value: 100% (95% CI 98.2 to 100)<br><br>Negative predictive value: 99.8% (95% CI 99.4 to 100)<br><br>Overall accuracy: 99.8% (calculated by CRD)	Patient selection: Low<br><br>Index test: Unclear<br><br>Reference standard: Low<br><br>Flow/timing: Unclear

section and Lumbar puncture section				Prevalence: 2.7% (14/510 who had CT)<br><br>**LP after negative CT (SAH):**<br><br>Sensitivity: 100% (95% CI 93.7 to 100)<br><br>Specificity: 96.8% (95% CI 94.8 to 98.8)<br><br>Positive predictive value: 9.1% (95% CI 0 to 26.1)<br><br>Negative predictive value: 100% (95% CI 99.5 to 100)<br><br>Overall accuracy: 96.8% (calculated by CRD)<br><br>Prevalence: 0.3% (1/309 who had LP)<br><br>CT was positive for SAH in 13 patients; 6 had an underlying lesion on angiography and 7 had perimesencephalic SAH. 4 CT scans were initially reported as 'normal' making patients eligible for LP, only to be subsequently altered in 3 cases to SAH positive after	

				neuroradiological interpretation of the CT scan.<br><br>LP was positive for SAH in 11 patients; 10 patients were LP positive but angiography negative (false positives).<br><br>**Diagnostic tests performed**<br><br>510 (98.6%) patients had a CT scan and 309 had LP. 491 patients were eligible for LP (490 initially negative on CT + 1 patient who went straight to LP without CT); 182 eligible patients did not have LP due to procedure failure (n=18), patient refusal or contraindication (n=65) or decision of attending doctor (n=99).<br><br>**Other significant diagnoses**<br><br>CT was positive for other significant aetiology in a further 14 patients: 4 cerebral infarction, 2 venous sinus thrombosis, 2 incidental cerebral aneurysm, 1 arachnoid cyst, 1 metastatic disease, 1 haemangioma, 1 subdural haemorrhage, 1 meningioma, 1 bleed into	

				glioblastoma. LP was positive for other significant aetiology in a further 17 patients: 16 viral meningitis and 1 nonocclusive sagital sinus thrombosis.	
Blok, 2015[35]<br><br>Retrospective cohort study<br><br>Emergency Departments at eleven non-academic hospitals, Netherlands<br><br>*Also reported in Pathway of CT followed by LP section*	760 neurologically intact (GCS 15) 'spontaneous' acute headache patients with suspected SAH, who underwent CT within 6 hours of onset (judged negative by radiologist) and subsequent LP.<br><br>Patient recruitment: January 2007 – January 2013.	CT (third generation scanner) <6 hours from headache onset (assessed by a staff radiologist), followed by LP >12 hours after onset (CSF was analysed using spectrophotometry).	Review of admission CTs in patients with bilirubin positive CSF by two neuroradiologists and one stroke neurologist. Lumbar puncture >12 hours after onset (CSF was analysed using spectrophotometry using a number of methods across the 11 sites: oxyhaemoglobin/bilirubin concentration, UK NEQAS, qualitative assessment of absorption curve, Leiden method, and bilirubin excess).	**Diagnostic accuracy results**<br><br>52 (7%) CSF samples were initially considered positive for SAH, but only one CT was positive for subarachnoid blood (in the basal cisterns) on review by two neuroradiologists and one stroke neurologist; angiography did not identify an aneurysm and the patient was diagnosed with non-aneurysmal perimesencephalic haemorrhage (with a benign clinical course and no readmission for SAH during 26 month follow-up). No subarachnoid blood was identified in the other 51 patients with positive CSF findings. 28/51 patients had angiography; aneurysm was identified in 8 patients (3 previously coiled). In those with an aneurysm it was considered that aneurysm rupture was unlikely and the aneurysm was considered incidental (4 were treated and 4 were not).	Patient selection: Low<br><br>Index test: Low<br><br>Reference standard: Unclear<br><br>Flow/timing: Unclear

				The negative predictive value for detection of subarachnoid blood on CT by staff radiologists working in a non-academic hospital was 99.9% (95% CI 99.3 to 100). SAH prevalence was 0.13% (1/760).	
Austin, 2018[44]<br><br>Interim analysis of a retrospective cohort study<br><br>Emergency department at one academic hospital, UK	250 patients attending the ED with suspected SAH who underwent CT.<br><br>Patient recruitment: January – December 2016.	Interpretation of CT scans for SAH by emergency physicians (images were viewed on desktop screens).<br><br>Average timeframe from symptom onset to scan was 48 hours (range 2 – 288).	Interpretation of CT scans for SAH by neuroradiologists (images were viewed using dedicated high definition screens for interpretation).	**Significant diagnoses**<br><br>20 (8%) patients had SAH. A further 5 scans had other positive findings; 3 intracranial haemorrhage, 1 subdural haematoma, 1 venous sinus thrombosis.<br><br>**Diagnostic accuracy results**<br><br>**Emergency physician interpretation of CT (intracranial pathologies):**<br><br>Sensitivity: 84% (95% CI 63.9 to 95.5)<br><br>Specificity: 95% (95% CI 90.9 to 97.2)<br><br>Three scans showing subarachnoid blood and one case of venous sinus thrombosis were interpreted as negative by Emergency Physicians. There was no difference in false	Patient selection: Unclear<br><br>Index test: High*<br><br>Reference standard: Unclear<br><br>Flow/timing: Low<br><br>(limited reporting, as only a correspondence article was available)<br><br>*Bias was considered high due to interpretation of index test on desktop screens, rather than high definition screens, as per reference standard

				negative interpretation between registrars and consultants. Gold standard was the final neuroradiologist report; neuroradiologists used dedicated high definition screens for interpretation.<br><br>**Diagnostic tests performed**<br><br>69 patients (30.6%) were further investigated; 59 (26.2%) had LP (3 had a positive result).	
**Lumbar puncture (CSF analysis)**					
Migdal, 2015[48]<br><br>Retrospective cohort study<br><br>Emergency Department at one academic hospital, USA	245 non-traumatic headache patients who presented with 'worst ever' or thunderclap headache and underwent LP to evaluate for SAH after normal CT. The study included 302 patients in total, 245 of which were included in a subgroup analysis of patients with 'low risk clinical features', with normal mental status, no known aneurysm at the time of LP and no known	LP after normal CT (64-slice CT scanner, interpreted by board-certified radiologists).<br><br>Diagnosis of SAH on LP was defined as xanthochromia in the CSF or RBCs >1x10^6/mm^3 in the final tube with aneurysm or arteriovenous malformation subsequently identified on cerebral angiography.	Not applicable.	There were no cases of SAH in the low risk subgroup. 13/245 (5.3%) of these patients had LP-related complications.<br><br>2/302 (0.66%) patients in the full population had SAH diagnoses based on LP; both had high-risk characteristics for SAH (i.e. altered mental status or known aneurysm), but no signs of intracranial haemorrhage on CT. 18/302 (6%) had LP-related complications that resulted in a return visit to the ED or hospitalisation, including 12 patients with low-pressure headache (4	Unclear

	prior SAH (who met our inclusion criteria).<br><br>Patient recruitment: 1 July 2010 – 30 June 2013.			patients treated with a blood patch), 4 patients with severe LP site pain and 2 patients with contaminated CSF cultures. No patients had an infectious or haemorrhagic complication arising from LP.<br><br>32/302 (10.6%) patients in the full population had an alternative diagnosis identified from LP; 19 had viral meningitis, 5 had bacterial meningitis, 1 had chemical meningitis from recent contrast exposure.<br><br>Head CTA identified 22 aneurysms in the 100 patients tested from the full population (22%).	
Perry, 2015[49]<br><br>Sub-study of a prospective cohort study<br><br>Emergency Departments at	1739 non-traumatic, alert (GCS 15) headache patients (peaking within 1 hour) with suspected SAH and an initial negative CT scan. The analysis included the 641 patients with an abnormal LP result.	LP with CSF analysis (5 sites used visual inspection, 1 used spectrophotometry). Risk threshold based on concentration of RBCs in sample. Median time from headache onset to LP was 18 hours.	CT or xanthochromia or red blood cells in the final tube of CSF with aneurysm on cerebral angiography (digital subtraction, magnetic resonance, or CT) requiring neurovascular intervention or resulting in death.	641/1739 patients had an abnormal LP result (red blood cells in the final tube or xanthochromia). 15 of which had aneurysmal SAH; 7 cases were identified by presence of xanthochromia and 8 had abnormal erythrocyte count in CSF.<br><br>**Diagnostic accuracy results**	Patient selection: Low<br><br>Index test: High<br><br>Reference standard: Low<br><br>Flow/timing: Unclear

twelve academic centres, Canada	Patient recruitment: November 2000 – December 2009 (appears to be patient overlap with Perry, 2011[43]).			**RBC count**<br><br>Optimal RBC count cut-off to differentiate traumatic tap from SAH was ≤2000x10^6/L. Sensitivity was 93.3% (95% CI 66.0 to 99.7) and specificity was 92.8% (95% CI 90.5 to 94.6%) at this cut-off.<br><br>**Visual inspection of xanthochromia**<br><br>Visual inspection of xanthochromia had sensitivity of 46.7% (95% CI 22.3 to 72.6) and specificity of 97.3% (95% CI 95.6 to 98.4).<br><br>**Risk classification based on threshold of <2000 x 10^6/L RBC and no xanthochromia (aneurysmal SAH):**<br><br>Sensitivity: 100% (95% CI 74.7 to 100)<br><br>Specificity: 91.2% (calculated by CRD)<br><br>Positive predictive value: 21.4% (95% CI 12.9 to 33.2)	

				Negative predictive value: 100% (95% CI 99.2 to 100)<br><br>Overall accuracy: 91.4% (calculated by CRD)<br><br>Prevalence: 2.3% (15/641)	
Dupont, 2008[46]<br><br>Retrospective cohort study<br><br>Emergency department at one academic medical centre, USA	152 non-traumatic, alert, neurologically intact (GCS 15) thunderclap headache patients (sudden and severe headache with maximal intensity at onset) with normal results on non-contrast CT. Mean time from headache onset to CT was 29.5 hours (range 1 hour to 10 days). Interpretation of CT results was performed by a radiologist or neuroradiologist.<br><br>Patient recruitment: 1 January 1998 – 1 January 2008.	LP with CSF analysis. CSF analysis of cell count, protein, glucose content and appearance was conducted in the hospital laboratory facility. Xanthochromia was determined by visual inspection of centrifuged samples on a background of white paper and under full-spectrum light. Mean time from headache onset to CSF analysis was 35.9 hours (range 2 hours to 10 days). Results were reported to the treating physician within 90 minutes of the LP procedure.	Four-vessel catheter angiography was performed in all patients with xanthochromic CSF (n=18). If no aneurysm was detected, the procedure was performed again within 7-14 days. Patients with an unruptured aneurysm, deemed to be an incidental finding, were noted.<br><br>Patients with non-xanthochromic CSF (n=99) and patients who refused LP (n=35) were followed up clinically.<br><br>A magnetic resonance angiographic study (1.5 T, gadolinium-enhanced) was performed in patients who were initially discharged from the ED but returned with	**Diagnostic accuracy results**<br><br>**CSF xanthochromia (cerebral aneurysm):**<br><br>Sensitivity: 93%<br><br>Specificity: 95%<br><br>Positive predictive value: 72%<br><br>Negative predictive value: 99%<br><br>Overall accuracy: 94.9% (calculated by CRD)<br><br>CSF xanthochromia was present in 18/117 (15%) patients who underwent LP; 13/18 (72%) had a ruptured cerebral aneurysm detected. 3/5 (60%) patients in whom aneurysm was not detected had a history of migraine, vs 2/13 (15%) of those with aSAH.	Patient selection: Low<br><br>Index test: Low<br><br>Reference standard: Low<br><br>Flow/timing: Low

			symptoms of a second sudden-onset headache (n=35).	Of the 99 patients without xanthochromia detected in the CSF, 35/99 (35%) underwent additional MR angiography on recurrence of their headaches; all were negative. 98/99 (99%) had no bleeding event at clinical follow-up. However, 1/99 (1%) patient who tested negative for xanthochromia was subsequently found to have a ruptured middle cerebral artery aneurysm (false negative result); this patient had (negative) CT performed 6 hours after headache onset and LP performed 9 hours after headache onset – whilst CSF was not deemed xanthochromic, the CSF RBC count remained between 20,000 and 30,000/µL in 4 successive collection tubes.<br><br>Patients with aneurysm had significantly higher red blood cell counts (mean 85,779 [SD 43,245]/µL) than patients without aneurysm (mean 98.7 [SD 646.2]/µL); p<0.001. Patients with aneurysm also had significantly higher total nucleated blood cell counts (mean 64.7 [SD 49.7]/µL)	

				than patients without aneurysm (mean 1.47 [SD 1.18]/µL); p=0.02.<br><br>152 (100%) patients had a negative CT scan and 117 (77%) underwent LP. 23% patients refused LP despite strong recommendations (none of which had bleeding events at clinical follow-up).<br><br>Prevalence: 9.2% (14/152 of the total cohort; calculated by CRD)	
Sansom, 2014[50]<br><br>Retrospective cohort study<br><br>Emergency department at one teaching hospital, UK	60 thunderclap headache patients with a negative CT scan result (mean time from headache onset to CT was 32.1 hours, range 2-170). 323 patients presented with thunderclap headache during the recruitment period, only the 60 patients who had a negative CT result and underwent LP were included in the analysis.	LP with CSF analysis (national guidelines for CSF analysis for xanthochromia were used).	Not applicable.	None of the 60 cases of thunderclap headache with negative CT were positive for xanthochromia.<br><br>52/60 CSF examinations were normal for all CSF parameters (protein, glucose, cells, microscopy and xanthochromia). 5 of 8 abnormal examinations were positive for oxyhaemoglobulin; 3 were associated with mild pleocytosis (<10 WBC x 10^6/L). Cerebral infarction was confirmed in 2 of the 8 patients with subsequent scans. CSF examination showed pleocytosis in the remaining case. Aneurysm was excluded in 5 patients with vascular imaging.	High<br><br>(limited reporting, as only a conference poster was available)

	Patient recruitment: 1 May 2013 – 31 October 2013.			Prevalence of SAH in the full population was 5.6% (18/323).	
Horstman, 2012[47]<br><br>Retrospective cohort study<br><br>Emergency department at one university hospital, Netherlands	30 patients with sudden severe headache or neck pain and negative head CT but bilirubin detected in CSF. WFNS score of 1 in all but one patient (WFNS 2, equivalent to 13-14 on GCS).<br><br>Patient recruitment: 2002 – 2007.	Bilirubin in the CSF (>0.05 at wavelength 458 nm). CSF was protected from light by wrapping in foil, then centrifuged at 1,500 rpm for 10 minutes. The supernatant was stored at 4◦C until analysis. CSF investigations were performed using a Beckman DU 650 spectrophotometer (Beckman Coulter, The Netherlands).	Not applicable.	Aneurysms were detected in 13/30 (43%) patients with bilirubin in their CSF, all of whom presented between 4 and 14 days after symptom onset. CT scans from patients from outside hospitals referred to our hospital were judged as normal by the radiologist at the outside hospital, but slight abnormalities were found in 4/30 (13.3%) after revision by the neuroradiologist at our hospital; 2 were positive for SAH, 2 were ambiguous (suspicion of small amount of blood in the pentagon).<br><br>Aneurysms were treated by coiling in 9 patients and clipping in 2; 2 patients were not treated due to poor clinical condition or refusal of further tests. 2/13 patients died within 3 months; 1 due to a re-bleed, the other due to secondary ischaemia. One further SAH patient had a poor outcome with major neurological deficits because of secondary ischeaemia.	Low

				All patients without an aneurysm detected were alive after 2-7 years of follow-up with no further SAH episodes.	
Cooper, 2016[36]<br><br>Retrospective cohort study<br><br>Clinical Decision Unit at one teaching hospital, UK<br><br>*Also reported in Pathway of CT followed by LP section and CT scan section*	517 non-traumatic, neurologically pristine (GCS 15) patients with acute sudden onset severe headache managed on a CDU pathway for exclusion of SAH.<br><br>Patient recruitment: January 2004 – December 2006.	CDU pathway of CT followed by LP.<br><br>Initial and verified non-contrast CT reports (performed on third-generation scanners) and LP results (all taken >12 hours from the index headache).	CT (verified by a consultant radiologist), LP (CSF positive for bilirubin on spectrophotometry or a uniformly blood-stained CSF sample across four bottles and positive angiography). If CT/LP strategy was not completed, sudden death or subsequent SAH was assessed at 12 months by analysing attendance and investigations (electronic hospital database).	**Diagnostic accuracy results**<br><br>**CT (SAH):**<br><br>Sensitivity: 92.9% (95% CI 79.5 to 100)<br><br>Specificity: 100% (95% CI 99.6 to 100)<br><br>Positive predictive value: 100% (95% CI 98.2 to 100)<br><br>Negative predictive value: 99.8% (95% CI 99.4 to 100)<br><br>Overall accuracy: 99.8% (calculated by CRD)<br><br>Prevalence: 2.7% (14/510 who had CT)<br><br>**LP after negative CT (SAH):**<br><br>Sensitivity: 100% (95% CI 93.7 to 100)	Patient selection: Low<br><br>Index test: Unclear<br><br>Reference standard: Low<br><br>Flow/timing: Unclear

				Specificity: 96.8% (95% CI 94.8 to 98.8)<br><br>Positive predictive value: 9.1% (95% CI 0 to 26.1)<br><br>Negative predictive value: 100% (95% CI 99.5 to 100)<br><br>Overall accuracy: 96.8% (calculated by CRD)<br><br>Prevalence: 0.3% (1/309 who had LP)<br><br>CT was positive for SAH in 13 patients; 6 had an underlying lesion on angiography and 7 had perimesencephalic SAH. 4 CT scans were initially reported as 'normal' making patients eligible for LP, only to be subsequently altered in 3 cases to SAH positive after neuroradiological interpretation of the CT scan.<br><br>LP was positive for SAH in 11 patients; 10 patients were LP positive but angiography negative (false positives).	

				**Diagnostic tests performed**<br><br>510 (98.6%) patients had a CT scan and 309 had LP. 491 patients were eligible for LP (490 initially negative on CT + 1 patient who went straight to LP without CT); 182 eligible patients did not have LP due to procedure failure (n=18), patient refusal or contraindication (n=65) or decision of attending doctor (n=99).<br><br>**Other significant diagnoses**<br><br>CT was positive for other significant aetiology in a further 14 patients: 4 cerebral infarction, 2 venous sinus thrombosis, 2 incidental cerebral aneurysm, 1 arachnoid cyst, 1 metastatic disease, 1 haemangioma, 1 subdural haemorrhage, 1 meningioma, 1 bleed into glioblastoma. LP was positive for other significant aetiology in a further 17 patients: 16 viral meningitis and 1 nonocclusive sagital sinus thrombosis.	

Valle Alonso, 2018[40]<br><br>Retrospective cohort study<br><br>Emergency Department at one regional hospital, Spain<br><br>*Also reported in Pathway of CT followed by LP section and CT scan section*	85 non-traumatic, sudden headache patients (peaking within 1 hour) without unconsciousness or neurological focus, presenting to the ED within 6 hours of symptom onset.<br><br>Patient recruitment: March 2012 – March 2013.	CT (within 6 hours) followed by LP, if CT negative for SAH.<br><br>The CT used was multi-slice (4-320 slices/rotation) with slices of 5 - 7.5 mm for the brain and 2.5 – 5 mm for the posterior fossa. The CT report was made by deputies of the radiology service, with over 5 years of experience and in consultation with the neuroradiologist when there was doubt.	LP was performed in all patients with a negative CT scan. Clinical follow-up at 6 months using medical records or phone calls where there was no conclusive data in medical records.	**Diagnostic accuracy results**<br><br>**CT within 6 hours (SAH):**<br><br>Sensitivity: 100% (calculated by CRD)<br><br>Specificity: 98.7% (calculated by CRD)<br><br>Positive predictive value: 90.9% (calculated by CRD)<br><br>Negative predictive value: 100% (calculated by CRD)<br><br>Overall accuracy: 98.8% (calculated by CRD)<br><br>Prevalence: 11.8% (calculated by CRD)<br><br>**Diagnostic tests performed**<br><br>74 (87%) patients underwent LP; LP was positive in 1 patient and inconclusive in 2 patients. However, bleeding was ruled out with later images; thus no cases of SAH were identified by LP. No cases of SAH were reported during the 6 months of follow-up. 7 patients experienced post puncture headache, going back to the ED and admission was	Patient selection: Unclear<br><br>Index test: Low<br><br>Reference standard: Low<br><br>Flow/timing: Low

				necessary for 2 of them for pain control.<br><br>**Other significant diagnoses**<br><br>The most frequent final diagnosis was migraine (38.8%). 9.4% had a severe diagnosis, such as meningitis (4.7%) and reversible cerebral vasoconstriction syndrome (4.7%).<br><br>**SAH patient signs and symptoms**<br><br>SAH patients were more likely to arrive at ED by ambulance (p=0.010) and have occipital headache location (p=0.012). Among the clinical signs highlighted, the presence of syncope (p=0.036), neck pain or stiffness (p=0.010), photophobia (p=0.001), nausea or vomiting (p=0.000), as well as higher numbers of systolic (mean 153 vs 126) and diastolic blood pressure (mean of 100 vs 80) (p=0.000).	
Brunell, 2013[45]	453 patients over 10 years of age who underwent LP to exclude SAH, including 400 patients with thunderclap	LP with CSF analysis. An automated quantitative measurement of bilirubin in the CSF was used. The CSF and plasma bilirubin and CRP	Not applicable.	295/453 (65%) LPs resulted in completely normal CSF-analysis and 138 (30%) were pathological in a way that was deemed insignificant by the treating physician, e.g. very mild	Low

Retrospective cohort study<br><br>Emergency department or outpatient clinics in neurology or infectious diseases at one university hospital, Sweden	headache (88%) and 53 patients where the treating physician wanted to perform LP to exclude SAH (e.g. patients with previous SAH or cases of severe headache with unclear onset).<br><br>Patient recruitment: January 2009 – December 2011.	measurements were performed on a high-throughput automatic analyser: Abbott Architect c8000 (Abbott Laboratories, Illinois, USA). Above the cut-off 350 nmol/L the CSF-bilirubin determinations were regarded as positive. Hemoglobin in CSF was measured by spectrophotometry at a fixed wavelength 415 nm, on a Hitatch U-1100, utilising 0.040 arbitrary units (AU) as a cut-off. Samples are routinely protected from light before analysis.		pleocytosis or raised protein. 14 (3%) patients had an alternative diagnosis (most commonly aseptic meningitis) and 5 (1.1%) had SAH.<br><br>4/5 SAH patients presented with thunderclap headache and had non-aneurysmal SAH not requiring surgical intervention. The other patient had decreased level of consciousness and prior history of SAH; due to poor general condition no further investigations or treatment were performed. All patients with SAH detected by LP underwent LP >12 hours after headache onset and CT >6 hours after headache onset. One patient was not CT-negative, but underwent LP prior to CT, which demonstrated bleeding.<br><br>11/14 patients with an alternative diagnosis presented with thunderclap headache and all diagnoses were based on LP performed >12 hours after headache onset. 6 patients had normal neurological examination and 3 had only discrete signs of meningism. 2 patients received antiviral treatment for herpex	

				simplex virus, 12 had no treatment and all patients made a full recovery.<br><br>153/453 (34%) patients were admitted for their LP after negative CT, including patients admitted to await the 12 hour time limit or because time could not be spared to perform the LP in the ED (additional patients were admitted for medical reasons, e.g. pain relief).<br><br>All patients had a CT scan and LP.	
Gangloff, 2015[51] Some results also taken from duplicate report[65]<br><br>Retrospective cohort study<br><br>Emergency Department at one university hospital, Canada	706 non-traumatic, neurologically intact (GCS 15) acute headache patients with suspected SAH and an initial negative CT scan (Siemens Sensation 4 between 2003-2008 and Sensation 16 from 2008 onwards; CT scan read by a radiologist).<br><br>Patient recruitment: 2003-2009 (may be	Visual and spectrophotometric inspection of xanthochromia. LP was undertaken >12 hours after symptom onset in 466 patients (67.5%), median 13 hours.<br><br>Visual analysis was performed on fresh CSF by the technologist on duty, immediately after arrival to the laboratory. Spectrophotometry was performed after visual assessment, using a quartz	Angiography (catheter angiogram, CT-angiogram). To avoid misclassifying incidental aneurysm with a traumatic tap as aSAH, positive cases were further reviewed by two physicians using a standardised data collection sheet – in case of disagreement medical charts were sent to a neurosurgeon for a third opinion.	**Diagnostic accuracy results**<br><br>**UK NEQAS CSF analysis (aneurysmal SAH):**<br><br>Sensitivity: 100% (95% CI 47.8 to 100)<br><br>Specificity: 98.1% (95% CI 96.7 to 99.0)<br><br>Positive predictive value: 27.8% (calculated by CRD)<br><br>Negative predictive value: 100% (calculated by CRD)	Patient selection: Low<br><br>Index test: Low<br><br>Reference standard: Low<br><br>Flow/timing: Unclear

	patient overlap with Perry, 2011[43]).	cuvette compared against a blank made of ultra-pure water and scanned from 350 nm to 700 nm using a Cary100 spectrophotometer (Varian). Resulting scans were analysed using the UK NEQAS 2008 approach and the Hendrik Duiser iterative approach.	The study had a safety-net for possible missed SAH; it is the only neurosurgical referral centre covering more than half the province of Quebec, a false-negative patient would eventually be picked up on a follow-up visit or readmission, or in the event of any sudden death through coroner investigation.	Overall accuracy: 98.2% (calculated by CRD)<br><br>Prevalence: 0.7%<br><br>13 (1.8%) false positive results; 9 of which had non-aneurysmal SAH.<br><br>**Iterative spectrophotometry method (aneurysmal SAH):**<br><br>Sensitivity: 100% (95% CI 47.8 to 100)<br><br>Specificity: 91.9% (95% CI 89.6 to 93.9)<br><br>56 (7.9%) false positive results; 18 of which were due to other indications (10 non-aneurysmal SAH, 7 meningitis, 1 hyperbilirubinemia disease).<br><br>**Visual xanthochromia (aneurysmal SAH):**<br><br>Sensitivity: 80% (95% CI 28.4 to 99.5)	

				Specificity: 98.7% (95% CI 97.5 to 99.4)<br><br>LP identified 5 aneurysmal SAH patients who had a negative CT; all had high red blood cell count (from 1310 to 63,000 x 10^6/L) and positive spectrophotometric xanthochromia; 4/5 were positive on visual inspection for xanthochromia. All 5 patients received coiling or clipping and had a good outcome.<br><br>4/5 SAH patients had delays longer than 24 hours prior to CT, the other patient received CT 2.5 hours after symptom onset.	
Perry, 2006[52]<br><br>Sub-study of a prospective cohort study<br><br>Emergency Departments at three tertiary care	220 non-traumatic, alert, neurologically intact (GCS 15) headache patients (peaking within 1 hour) or syncope associated with headache.<br><br>Patient recruitment: July 2002 – January 2004 (appears to be patient	LP with CSF examined using spectrophotometry (Milton Roy Spectronic 1001plus). After routine analysis for cell count and visible xanthochromia, any remaining CSF in the final tube was centrifuged and frozen for later spectrophotometry. Absorbances were measured across a 1-cm light path at 360 nm, 415 nm, 440 nm, 476 nm and 530 nm relative to a saline	CT, LP (xanthochromia on visual inspection or >5x10^6/L RBCs in the final tube of CSF with aneurysm or arteriovenous malformation seen on angiography) and clinical follow-up (telephone follow-up at 30 days).	**Diagnostic accuracy results**<br><br>**Visual inspection (SAH):**<br><br>Sensitivity: 50% (95% CI 3.0 to 81)<br><br>Specificity: 97% (95% CI 92 to 99)<br><br>**Traditional definition (SAH):**	Patient selection: Low<br><br>Index test: Low<br><br>Reference standard: Low<br><br>Flow/timing: Low

university hospitals, Canada	overlap with Perry, 2011[43] and Perry, 2015[49]).	blank. Four different definitions of positive spectrophotometry were selected a priori: Traditional, Chalmers and Kiley, Chalmers revised and UK NEQAS. The interval between headache onset and LP was >12 hours in 55% patients.<br><br>Comparator: Visual inspection of the centrifuged CSF for xanthochromia against a white paper background under full spectrum light.		Sensitivity: 100% (95% CI 16 to 100)<br><br>Specificity: 29% (95% CI 23 to 35)<br><br>**Chalmers and Kiley definition (SAH):**<br><br>Sensitivity: 0% (95% CI 0 to 16)<br><br>Specificity: 89% (95% CI 84 to 92)<br><br>**Chalmers revised definition (SAH):**<br><br>Sensitivity: 100% (95% CI 3.0 to 100)<br><br>Specificity: 29% (95% CI 23 to 35)<br><br>**UK NEQAS definition (SAH):**<br><br>Sensitivity: 100% (95% CI 3.0 to 100)<br><br>Specificity: 83% (95% CI 76 to 87)	

				Prevalence: 1 patient had aneurysmal SAH and 1 patient had an incidental unruptured aneurysm.<br><br>One patient with aneurysm had normal CT 8 hours after headache onset; LP demonstrated high levels of RBCs (53,500x10^6/L) and visible xanthochromia, with aneurysm (11x8mm) confirmed on CT angiography. The other patient had normal CT 3 days after headache onset; CSF contained RBCs (41x10^6/L) but no visual xanthochromia and was classed as traumatic tap by the treating physician. Aneurysm (5mm) was confirmed on CT angiography but was considered incidental and not treated; the patient remained well 1 year later.<br><br>**Diagnostic tests performed**<br><br>87.7% patients had a CT scan and 100% had LP. 5.9% patients had a CT angiogram. If presence of visible xanthochromia (visual inspection) were the only indication for angiography, the angiography rate would reduce by 85%. However,	

				using any of the 3 sensitive spectrophotometric definitions of xanthochromia would increase angiography rates from 254% to 1208% compared with current practice.	
Heiser, 2015[53]<br><br>Retrospective cohort study<br><br>Emergency departments at two academic hospitals, USA	676 non-traumatic, alert, acute headache patients who underwent LP to rule out SAH and had an abnormal result on CSF.<br><br>Patient recruitment: Not reported. 6 year study period.	Validation of a clinical prediction rule to differentiate between traumatic LP and SAH, based on CSF findings (RBC count >2000 x 10^6/L and the presence of xanthochromia, if neither criteria present, aSAH can be excluded).	SAH was confirmed in 49 patients using diagnostic imaging. Demographics, co-morbidity, clinical findings, diagnostic testing and final diagnosis were obtained from ED records. Unclear whether all patients had diagnostic testing and/or other reference standard.	**Diagnostic accuracy results**<br><br>**Clinical prediction rule (RBC count >2000 x 10^6/L and presence of xanthochromia) (SAH):**<br><br>Sensitivity: 81.6% (95% CI 68.0 to 91.2)<br><br>Specificity: 97.3% (95% CI 95.7 to 98.4)<br><br>Positive predictive value: 70.2% (calculated by CRD)<br><br>Negative predictive value: 98.5% (calculated by CRD)<br><br>Overall accuracy: 96.2% (calculated by CRD)<br><br>Prevalence: 7.2% (49/676)<br><br>The incidence of traumatic LP was 24.4%. The range of values in tube 4 for the SAH group was 120 to 521,500 RBCs, suggesting that there	Patient selection: Low<br><br>Index test: Unclear<br><br>Reference standard: Unclear<br><br>Flow/timing: Unclear<br><br>(limited reporting, as only a conference presentation was available)

				is not a CSF RBC cut-off value at which one can safely exclude SAH. We found no risk factor or combination of clinical factors that would improve ED provider sensitivity without markedly decreasing specificity.	
**CT Angiography**					
Alons, 2015[54]<br><br>Retrospective cohort study<br><br>Emergency department at one teaching hospital, Netherlands	70 non-traumatic, neurologically intact, acute severe headache patients with normal non-contrast CT (evaluated by specialised neuroradiologists) and CSF findings (all patients had CT and LP).<br><br>Patient recruitment: January 2008 – May 2011.	CT angiogram using GE Lightspeed 64-slice CT scanner. All but 1 scan was made within a week of the occurrence of the headache; 1 scan was made after 3 weeks. MRI was also used in 15 patients.	Not applicable.	There were no cases of SAH.<br><br>13/70 (19%) patients had a vascular abnormality identified on CTA; 8 (11%) had aneurysms (3 were coiled, 3 were clipped and 2 received follow-up CTA to monitor aneurysm size), 2 cerebral venous thrombosis, 2 reversible cerebral vasoconstriction syndrome and 1 patient had ischemia of the posterior circulation in the right occipital area.	Unclear
Alons, 2018[55]<br><br>Retrospective cohort study and meta-analysis	88 neurologically intact, acute headache patients (developing within 5 minutes and lasting ≥1 hour) with normal non-contrast CT and CSF findings, when performed (LP	CT angiography using Aquilion One (Toshiba Medical Systems), Aquilion 64 (Toshiba Medical Systems) or GE Lightspeed 64-slice CT scanners.	Not applicable.	There were no cases of SAH.<br><br>5/88 patients had a vascular abnormality identified on CTA; 1 aneurysm (a small unruptured aneurysm with a normal LP, not	Unclear

Emergency departments at two university affiliated secondary referral centres, Netherlands	performed in 35% patients). The meta-analysis also included 641 patients identified from the literature.<br><br>Patient recruitment: 2011 – 2014.			considered to be the cause of the headache), 1 cerebral venous thrombosis, 2 reversible cerebral vasoconstriction syndrome and 1 cervical dissection. The aneurysm was treated with clip ligation, the reversible cerebral vasoconstriction syndrome patients were followed up clinically and the other two patients were followed up with medication change.<br><br>1 patient experienced an adverse event associated with CTA; a short-term allergic reaction to iodinated contrast media.	
**History, examination and investigation**					
Locker, 2004[56]<br><br>Retrospective cohort study<br><br>Emergency department at one teaching hospital, UK	353 non-traumatic, neurologically intact (GCS ≥14) headache patients. 36/353 patients presented with 'first or worst' headache and normal neurological examination (who met our inclusion criteria).	Adequacy of history, examination and investigation (CT and LP).	Not applicable.	7/353 (2%) patients were diagnosed with SAH; 4 had abnormal neurological examination, 3 presented with 'first or worst' headache (3/36; 8.3%).<br><br>Other secondary headaches identified in the full study population were: 1 intracranial bleed, 8 cerebral/cerebellar infarct, 3 meningitis, 18 systemic infection, 28 'other' secondary headache. 280	Unclear

	Patient recruitment: 1 January 2000 – 31 December 2000.			patients were diagnosed with primary headaches (migraine, tension headache, cluster headache or 'other' primary headache). The final diagnosis was not known for 8 patients.<br><br>1 patient was re-admitted within 3 months with SAH, it is unclear whether this was originally missed or new.<br><br>4 characteristics were selected as predictors of secondary headache: age >65 years, temperature >38◦C, systolic BP >160 mmHg, presence of neck stiffness. The presence of at least one of these features in the study population predicted secondary headache with a sensitivity of 37.8% and a specificity of 82.1%.<br><br>Only 1 patient had an adequate history recorded and no patient had a complete examination recorded.	
Perry, 2005[57]	747 non-traumatic, alert, neurologically intact (GCS 15) acute headache	Patient assessment made by attending physicians certified in emergency medicine or	CT (3rd generation or higher, verified by a neuroradiologist), LP	50/747 (6.7%) patients had SAH. 7 patients (0.94%) had other serious illnesses; 4 CNS neoplasm, 2 other	Patient selection: Low

Prospective cohort study<br><br>Emergency departments at three university-affiliated tertiary care teaching hospitals, Canada	patients (peaking within 1 hour) or syncope associated with headache.<br><br>Patient recruitment: November 2000 – March 2003 (appears to be patient overlap with Perry, 2011[43]).	supervised residents in an emergency medicine training program (without the use of a clinical decision rule).	(xanthochromia on visual inspection or >5x10^6/L RBCs in the final tube of CSF with aneurysm or arteriovenous malformation seen on angiography).	type of cerebral haemorrhage, 1 bacterial meningitis. 71.8% were diagnosed as having benign headache or migraine.<br><br>The emergency physicians' pre-test probability that their patient had a SAH was assessed using a receiver operating characteristic (ROC) curve; the area under the ROC curve was 0.85 (95% CI 0.80 to 0.91) (data available for 639 cases). There were 3 SAH patients for whom the physician pre-test probability was ≤2%; these patients had perimesencephalic bleed (n=1), vasculitis with SAH (n=1) and a 4.5mm right superior hypophyseal artery aneurysm (n=1, although unclear whether the patient had an SAH or a benign headache with an incidental aneurysm – CT was normal and LP was equivocal). Using the pre-test probability of ≥2% as the threshold to use diagnostic tests for headache patients, the sensitivity of clinical suspicion was 93% (95% CI 81 to 97) and specificity was 49% (95% CI 45 to 53).	Index test: Low<br><br>Reference standard: Low<br><br>Flow/timing: Low

				Physicians reported being "uncomfortable" (47.3% cases) or "very uncomfortable" (28.1% cases) with performing no test in 75.4% of cases (data available for 659 cases) and being "uncomfortable" (37.6% cases) or "very uncomfortable" (12.0% cases) with performing LP without CT in 49.6% cases (data available for 625 cases).<br><br>79.9% patients had a CT scan and 45.9% had LP; 42.6% had CT and LP.	
Backes, 2015[58]<br><br>Retrospective cohort study<br><br>Emergency department at one university hospital, Netherlands	247 non-traumatic, alert, neurologically intact (GCS 15) headache patients (peaking within minutes and lasting ≥1 hour). Patients were identified from databases of SAH patients and patients in whom SAH was ruled out using CT and LP. Diagnostic accuracy results were presented for 223 patients, as information on neck stiffness was missing for 24 patients.	Neurologic examination for neck stiffness as a predictor of SAH. The time interval between symptom onset and neurological examination was dichotomised into ≤6 hours and 6-72 hours.	CT or presence of bilirubin at CSF absorption spectrophotometry.	114 (46%) patients had SAH; in 2 patients head CT was negative for SAH but CSF tested positive for bilirubin and aneurysm was confirmed using CT angiogram.<br><br>82 patients had neck stiffness at neurological examination, although this was mild or ambiguous for 18 of these patients.<br><br>**Diagnostic accuracy results**<br><br>**Neck stiffness (SAH):**	Patient selection: Low<br><br>Index test: Low<br><br>Reference standard: High<br><br>Flow/timing: High

	Patient recruitment: 1 January 2005 – 1 September 2013 (likely patient overlap with Backes, 2012[41]).			Sensitivity: 67.0% (95% CI 57.9 to 76.1)<br><br>Specificity: 89.2% (95% CI 83.6 to 94.7)<br><br>Positive predictive value: 84.1% (95% CI 74.4 to 91.3)<br><br>Negative predictive value: 75.9% (95% CI 68.8 to 82.9)<br><br>Overall accuracy: 78.9% (calculated by CRD)<br><br>Prevalence: 46%<br><br>**Neck stiffness assessed within 6 hours (SAH):**<br><br>Sensitivity: 59.5% (95% CI 47.4 to 70.7)<br><br>Specificity: 93.1% (95% CI 84.5 to 97.7)<br><br>Positive predictive value: 89.8% (95% CI 77.8 to 96.6)<br><br>Negative predictive value: 69.1% (95% CI 58.9 to 78.1)	

				**Neck stiffness assessed between 6-72 hours (SAH):**<br><br>Sensitivity: 86.2% (95% CI 68.3 to 96.1)<br><br>Specificity: 83.3% (95% CI 69.8 to 92.5)<br><br>Positive predictive value: 75.8% (95% CI 57.7 to 88.9)<br><br>Negative predictive value: 90.9% (95% CI 78.3 to 97.5)<br><br>The presence of neck stiffness at neurological examination was more strongly predictive of SAH in subgroups with other high-risk clinical characteristics such as being ≥40 years old, vomiting and transient loss of consciousness.	

Abbreviations: aSAH, aneurysmal subarachnoid haemorrhage; BP, blood pressure; CDU, Clinical Decision Unit; CI, confidence interval; CNS, central nervous system; CRD, Centre for Reviews and Dissemination; CSF, cerebrospinal fluid; CT, computed tomography; CTA, computed tomography angiography; ED, Emergency Department; GCS, Glasgow Coma Scale; LP, lumbar puncture; MRI, Magnetic resonance imaging; RBC, red blood cell; ROC, receiver operating characteristic; SAH, subarachnoid haemorrhage.
