# Supplementary material for: Management of patients presenting to the emergency department with sudden onset severe headache: systematic review of diagnostic accuracy studies
Source: Emerg Med J. 2022 Mar 31;39(11):818–25. doi: 10.1136/emermed-2021-211900 (PMC9613855; doi:10.1136/emermed-2021-211900)
Supplement: Supplementary data [file emermed-2021-211900supp003.pdf]

Supplementary File 3 Quality assessment results

Cohort/before and after studies assessed using QUADAS-2 (n=28)

Study	Study design	Risk of bias level of concern				Applicability level of concern		
		**Patient selection**	**Index test**	**Reference standard**	**Flow and timing**	**Patient selection**	**Index test**	**Reference standard**
Perry, 2010[25]	Prospective cohort study	Unclear	Low concern	Low concern	Low concern	Low concern	Low concern	High concern
Matloob, 2013[24]	Retrospective cohort study	Low concern	Unclear	Unclear	High concern	Low concern	Unclear	Low concern
MacDonald, 2012[23] (abstract)	Retrospective cohort study	Unclear	Unclear	Low concern	Unclear	Unclear	Unclear	Low concern
Kelly, 2014[22]	Retrospective cohort study	Low concern	High concern	Low concern	High concern	High concern	Unclear	Low concern
Perry, 2013[26]	Prospective cohort study	Low concern	Low concern	Low concern	Low concern	Low concern	Low concern	Low concern
Yiangou, 2017[27] (poster)	Retrospective cohort study	Unclear	Unclear	Low concern	Low concern	Low concern	Unclear	Low concern
Perry, 2017[32]	Prospective cohort study	Low concern	Low concern	Low concern	Low concern	Low concern	Low concern	Low concern
Bellolio, 2015[28]	Retrospective cohort study	Unclear	Unclear	Unclear	Low concern	Unclear	Unclear	Low concern
Wu, 2019[34]	Retrospective cohort study	Low concern	Unclear	High concern	High concern	Unclear	Unclear	Unclear
Chu, 2018[30]	Retrospective cohort study	Unclear	Unclear	Low concern	Low concern	Unclear	Unclear	Low concern
Pathan, 2018[31]	Retrospective cohort study	Low concern	Unclear	Low concern	Unclear	Low concern	Unclear	Low concern
Cheung, 2018[29]	Retrospective cohort study	Low concern	Unclear (Ottawa SAH Rule) High concern (modified Ottawa SAH Rule)	Low concern	Low concern	Low concern	High concern	Low concern
Perry, 2020[33]	Prospective before/after implementation study	Low concern	Low concern	Low concern	Low concern	Low concern	Unclear	Low concern

Study	Study design	Risk of bias level of concern				Applicability level of concern		
		**Patient selection**	**Index test**	**Reference standard**	**Flow and timing**	**Patient selection**	**Index test**	**Reference standard**
Perry, 2008[39]	Prospective cohort study	Low concern	Low concern	Low concern	Low concern	Low concern	High concern	Low concern
Valle Alonso, 2018[40]	Retrospective cohort study	Unclear	Low concern	Low concern	Low concern	Low concern	Low concern	Low concern
Cooper, 2016[36]	Retrospective cohort study	Low concern	Unclear	Low concern	Unclear	Low concern	Low concern	Low concern
Blok, 2015[35]	Retrospective cohort study	Low concern	Low concern	Unclear	Unclear	High concern	Low concern	Low concern
Khan, 2017[42]	A priori planned secondary analysis of two sequential prospective cohort studies	Low concern	Low concern	Low concern	Low concern	Low concern	Low concern	High concern
Perry, 2011[43]	Prospective cohort study	Low concern	Low concern	Low concern	Low concern	Low concern	Low concern	High concern
Backes, 2012[41]	Retrospective cohort study	Low concern	Low concern	Low concern	Low concern	High concern	Unclear	Low concern
Austin, 2018[44]	Retrospective cohort study	Unclear	High concern (index test was interpreted on inferior screens to reference standard)	Unclear	Low concern	High concern	Unclear	Unclear
Perry, 2015[49]	Sub-study of a prospective cohort study	Low concern	High concern	Low concern	Unclear	High concern	High concern	High concern
Dupont, 2008[46]	Retrospective cohort study	Low concern	Low concern	Low concern	Low concern	Low concern	High concern	Low concern
Gangloff, 2015[51]	Retrospective cohort study	Low concern	Low concern	Low concern	Unclear	Unclear	Low concern	Low concern
Perry, 2006[52]	Sub-study of a prospective cohort study	Low concern	Low concern	Low concern	Low concern	High concern	High concern	High concern

Study	Study design	Risk of bias level of concern				Applicability level of concern		
		**Patient selection**	**Index test**	**Reference standard**	**Flow and timing**	**Patient selection**	**Index test**	**Reference standard**
Heiser, 2015[53] (presentation)	Retrospective cohort study	Low concern	Unclear	Unclear	Unclear	Unclear	Unclear	Unclear
Perry, 2005[57]	Prospective cohort study	Low concern	Low concern	Low concern	Low concern	Low concern	Unclear	Low concern
Backes, 2015[58]	Retrospective cohort study	Low concern	Low concern	High concern	High concern	High concern	Unclear	Low concern
Total	6 prospective cohort studies<br>18 retrospective cohort studies<br>1 before/after study<br>1 secondary analysis<br>2 sub-studies	21 low concern<br>7 unclear<br>0 high concern	15 low concern<br>10 unclear<br>3 high concern	21 low concern<br>5 unclear<br>2 high concern	17 low concern<br>7 unclear<br>4 high concern	15 low concern<br>6 unclear<br>7 high concern	9 low concern<br>14 unclear<br>5 high concern	20 low concern<br>3 unclear<br>5 high concern

Cohort/before and after studies not eligible for QUADAS-2 (n=9)

Study	Study design	Clearly defined inclusion criteria	Representative sample*	Groups similar at baseline	Clearly described & consistent delivery of intervention*	Reliable and consistent outcome assessment*	Blinded outcome assessment	Outcome data complete/attrition low*	Adequate follow-up duration*	Overall judgement of risk of bias+
Perry, 2002[38]	Retrospective cohort study	Yes	Yes	N/A	Unclear	Unclear	N/A	Yes	Yes	Unclear
Dutto, 2009[37]	Before and after study	Yes	Yes	Yes	Yes	Unclear	N/A	Yes	Yes	Unclear
Migdal, 2015[48]	Retrospective cohort study	Yes	Yes	N/A	Yes	Yes	N/A	Yes	Unclear	Unclear
Sansom, 2014[50] (poster)	Retrospective cohort study	Unclear	Yes	N/A	Unclear	Unclear	N/A	No	Unclear	High
Horstman, 2012[47]	Retrospective cohort study	Yes	Yes	N/A	Yes	Yes	N/A	Yes	Yes	Low
Brunell, 2013[45]	Retrospective cohort study	Yes	Yes	N/A	Yes	Yes	N/A	Yes	Yes	Low
Alons, 2015[54]	Retrospective cohort study	Yes	Yes	N/A	Yes	Yes	N/A	Yes	Unclear	Unclear
Alons, 2018[55]	Retrospective cohort study	No	Unclear	N/A	Yes	Yes	N/A	Yes	Unclear	Unclear
Locker, 2004[56]	Retrospective cohort study	Yes	Yes	N/A	Unclear	Yes	N/A	Yes	Yes	Unclear

* Key domains.

+ Each study was given an overall risk of bias judgement; studies that had a low risk of bias for all key domains were judged to have a low overall risk of bias, studies that had a high risk of bias for one or more key domains were judged to have a high overall risk of bias, and studies that had an unclear risk of bias (and no high risk of bias) for one or more key domains were judged to have an unclear overall risk of bias.

Abbreviations: N/A, not applicable.
